# A kit-based aluminium-[^18^F]fluoride approach to radiolabelled microbubbles†

**DOI:** 10.1039/d1cc04790f

**Published:** 2021-10-15

**Authors:** Jin Hui Teh, Marta Braga, Louis Allott, Chris Barnes, Javier Hernández-Gil, Meng-Xing Tang, Eric O. Aboagye, Nicholas J. Long

**Affiliations:** Department of Chemistry, Molecular Sciences Research Hub, Imperial College London UK n.long@imperial.ac.uk; Department of Surgery & Cancer, Imperial Centre for Translational and Experimental Medicine, Imperial College London UK eric.aboagye@imperial.ac.uk; Positron Emission Tomography Research Centre, Faculty of Health Sciences, University of Hull UK; Department of Bioengineering, Imperial College London UK

## Abstract

The production of ^18^F-labelled microbubbles (MBs) *via* the aluminium-[^18^F]fluoride ([^18^F]AlF) radiolabelling method and facile inverse-electron-demand Diels–Alder (IEDDA) ‘click’ chemistry is reported. An [^18^F]AlF-NODA-labelled tetrazine was synthesised in excellent radiochemical yield (>95% RCY) and efficiently conjugated to a *trans*-cyclooctene (TCO) functionalised phospholipid (40–50% RCY), which was incorporated into MBs (40–50% RCY). To demonstrate the potential of producing ^18^F-labelled MBs for clinical studies, we also describe a kit-based approach which is amenable for use in a hospital radiopharmacy setting.

## Abstract 2

A facile, kit-based method for ^18^F-labelling of ultrasound microbubble contrast agents is reported using the IEDDA ligation between a *trans*-cyclooctene modified phospholipid and a [^18^F]AlF-tetrazine tracer, enabling *in vivo* tracking of microbubbles.

## Graphical abstract

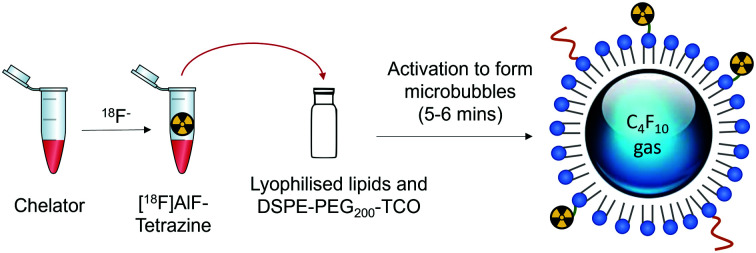

Microbubbles (MBs) are widely used to enhance ultrasound (US) contrast in echocardiography, characterise lesions, and evaluate perfusion.^1^ These ultrasound contrast agents consist of a gas core encapsulated by a stabilising shell, usually made up of phospholipids, proteins, or polymers. With a size of 1–5 μm, MBs are restricted to the vasculature, but recent developments in targeting ligands have enabled these contrast agents to image diseases at a molecular level.^2^ In this regard, the conjugation of a targeting ligand to the microbubble shell enables active targeting of angiogenesis,^3,4^ inflammation,^5^ thrombosis,^6^ and tumours.^7,8^ A phospholipid-based formulation, BR55, has also shown promising results recently for detecting various cancer types during human trials.^9,10^

Despite these advances, clinical translation of these targeted microbubbles remains challenging, partly due to the localised nature of ultrasound imaging.^11^ This makes it difficult to monitor the biodistribution and pharmacokinetics of new microbubble formulations.

To overcome this, several groups have developed dual modal positron emission tomography/ultrasound (PET/US) MB formulations to allow the assessment of microbubble biodistribution, making use of the high sensitivity and penetration depth of PET.^12–14^ However, two of these formulations use streptavidin–biotin interactions for the incorporation of the fluorine-18 (^18^F) isotope, making them unsuitable for human use due to their immunogenicity.^13,14^ Promisingly, Ferrara *et al.* radiolabelled a lipid molecule by ^18^F-nucleophilic substitution, thus eliminating the need for streptavidin–biotin interactions.^12^ However, the radiolabelled lipid was purified in hexane and evaporated in a nitrogen stream; this is a challenging procedure to implement for the production of clinical-grade radiopharmaceuticals, and the toxicity of the solvent requires rigorous quality control validation to ensure its absence from radiopharmaceutical formulation. Expertise in organic ^18^F-fluorination chemistry and specialist production facilities are also required to produce these MBs, presenting a potential obstacle to their clinical and routine use.^15^

To ameliorate these concerns, our group recently reported a convenient radiometal chelation approach, in combination with inverse electron demand Diels Alder (IEDDA) chemistry, to radiolabel MBs using gallium-68 (^68^Ga, *t*_1/2_ = 68 min, *E*_β+, max_ = 1.9 MeV).^16^ Although the microbubbles can be produced within 50 minutes from ^68^Ge/^68^Ga generator elution, the relatively short half-life of ^68^Ga, and somewhat onerous manual radiosynthesis process, reduce opportunities for further functionalisation (*i.e.* with targeting moieties). Furthermore, the limited production capacity of ^68^Ge/^68^Ga-generator reduces the number of patient doses from one elution of the generator, resulting in lower patient throughput.^17^

To overcome these challenges, and to improve the accessibility of ^18^F-labelled MBs over previous studies, we designed a convenient and facile ^18^F-microbubble labelling method using aluminium-[^18^F]fluoride ([^18^F]AlF) radiochemistry. The [^18^F]AlF method reported by McBride *et al.* combines the favourable decay characteristics of ^18^F (*t*_1/2_ = 110 min, *E*_β+, max_ = 0.64 MeV), with the convenience of metal-based radiolabelling.^17–23^

In brief, the aluminium-[^18^F]fluoride ([^18^F]AlF) method was used to radiolabel a tetrazine-containing prosthetic group (PG) and subsequently conjugated to *trans*-cyclooctene (TCO) functionalised lipids *via* the rapid IEDDA reaction (Fig. 1).

**Fig. 1:**
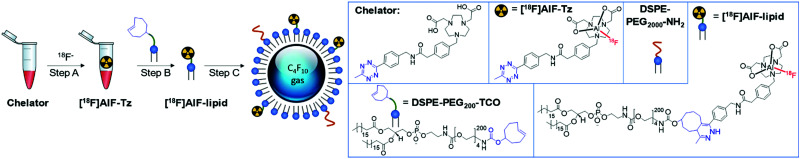
Schematic representation of ^18^F-labelling of microbubbles developed in this study, where radiolabelling is achieved by reaction of a TCO-containing lipid (DSPE-PEG_200_-TCO) and a tetrazine-functionalised ^18^F prosthetic group. (Step A): ^18^F-labelling of chelator (>95% RCY, RCP); (step B): IEDDA reaction of ^18^F-labelled chelator and TCO-lipid (40–50% RCY); (step C): formation of [^18^F]AlF-microbubbles (40–50% RCY).

A lipid-based microbubble formulation was chosen due to its versatility and widespread application.^16,24^ Dipalmitoyl phosphatidylcholine (DPPC) and dipalmitoyl phosphate (DPPA), which are biocompatible surfactants used in drug delivery vehicles,^25^ form the bulk of the stabilising microbubble shell; whereas DSPE-PEG_2000_-NH_2_ stabilises microbubbles from coalescence, increases circulation time, and acts as a handle for ligand conjugation.^26^ Using a formulation of 75 : 10 : 10 : 5 mol% DPPC : DPPA : DSPE-PEG_200_-TCO : DSPE-PEG_2000_-NH_2_, microbubbles were produced with precise concentration and size profiles (Fig. 2). This was in agreement with our previous formulation,^16^ as confirmed by optical microscopy and zeta potential measurements. With the TCO functionality successfully incorporated into the microbubbles, this formulation was carried forward for ^18^F-labelling.

**Fig. 2:**
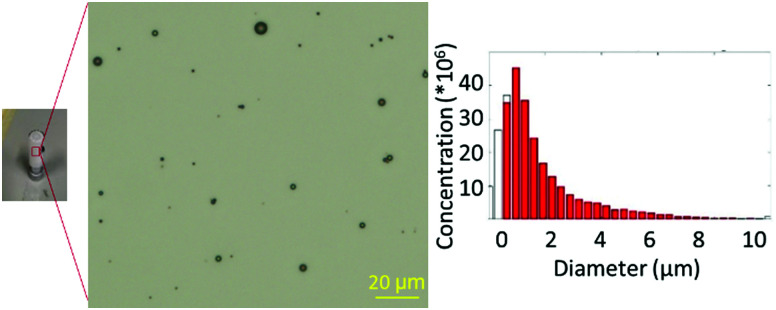
Left: Optical microscopy image of microbubbles; right: size distribution of microbubbles.

A tetrazine-functionalised 1,4,7-triazacyclononane-1,4-diacetate (NODA) chelator was synthesised by amide coupling tetrazine (**1**) and NODA-methylphenyl acetic acid (**2**) to produce (**3**) (Scheme 1). The NODA macrocycle was selected for this study because pentadentate chelators radiolabel more efficiently in higher radiochemical yield (RCY) than hexadentate chelators, where the free carboxylate arm competes with ^18^F^−^ for the final Al coordination site.^19,27^

**Scheme 1:**
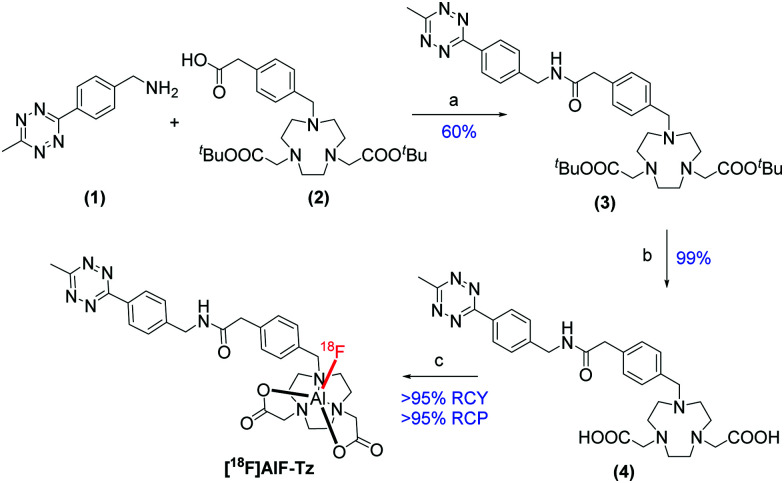
Synthesis of [^18^F]AlF-Tz. Reaction conditions: (a) HBTU, Et_3_N, DMF, 40 °C, 24 h; (b) TFA, CH_2_Cl_2_, r.t., 24 h; (c) AlCl_3_, [^18^F]F^−^, 0.5 M sodium acetate : MeCN 2 : 3 v : v, pH 4.2, 100 °C, 20 min.

Upon deprotection of (**3**) to reveal the carboxylate arms, NODA-tetrazine (**4**) was radiolabelled with [^18^F]AlF in >95% RCY and >95% radiochemical purity (RCP) after optimisation (Tables S1–S3, ESI†). The product identity and reaction efficiency were determined by radio-HPLC and radio-TLC (Fig. S3, ESI†). [^18^F]AlF-Tz was purified by using a solid phase extraction (SPE) cartridge (Oasis Prime HLB), and used in subsequent IEDDA reactions with DSPE-PEG_200_-TCO. In total, [^18^F]AlF-Tz was synthesised and purified in under 30 min.

Decomposition of [^18^F]AlF-Tz occurred in EtOH at rate of 3–5% per hour (Table S5, ESI†). Although this phenomena was observed in similar [^18^F]AlF-tetrazine molecules, presumably due to decomposition of the tetrazine moiety, the effect on the tetrazine–TCO conjugation is minimal if the reaction is carried out promptly after isolation of the [^18^F]AlF-tetrazine.^28,29^

Following successful isolation of [^18^F]AlF-Tz, the efficiency of the tetrazine–TCO conjugation reaction was examined. A purified fraction of [^18^F]AlF-Tz in EtOH was conjugated to an equimolar quantity of DSPE-PEG_200_-TCO, resulting in 40–50% conversion after heating at 60 °C for 20 min (non-isolated product, determined by radio-HPLC, Fig. 3). The appearance of a new peak with longer retention time (*R*_t_ = 7 : 35 min : s) than the precursor (*R*_t_ = 1 : 29) corresponds to the formation of the [^18^F]AlF-lipid, which was also confirmed by radio-TLC (Fig. S4, ESI†).

**Fig. 3:**
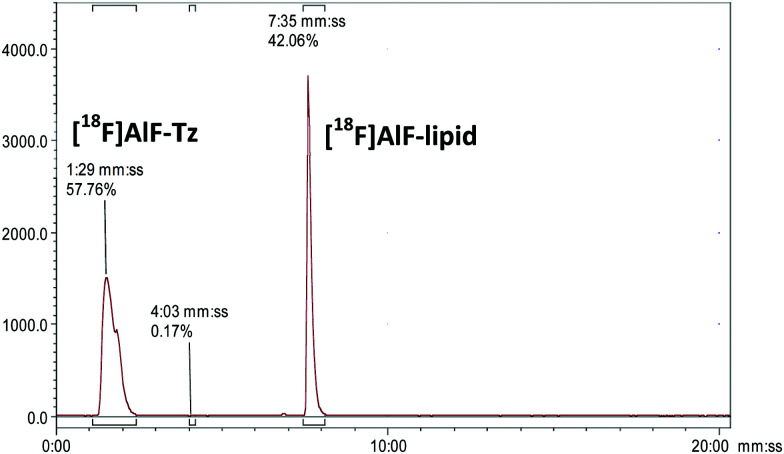
Radio-HPLC chromatogram showing the formation of [^18^F]AlF-lipid (*t*_R_ = 7 : 25 mm : ss) from [^18^F]AlF-Tz (*t*_R_ = 1 : 29 mm : ss) *via* an IEDDA “click” reaction.

Direct labelling of phospholipids by [^18^F]AlF was not investigated due to lipid hydrolysis under the acidic conditions necessary for [^18^F]AlF radiolabelling (pH 4–5),^30^ and the irreversible retention of these lipid compounds on a range of cartridges.^16^

Next, the [^18^F]AlF-lipid was incorporated into MBs together with DPPC, DPPA and DSPE-PEG_2000_-NH_2_. Unreacted [^18^F]AlF-Tz and remaining free lipids were removed by a centrifugal purification methodology.^16^ Once centrifuged, microbubbles were collected as a concentrated layer of foam at the top of the vial, and unincorporated components remained in the infranatant. This generated [^18^F]AlF-MBs (48 ± 12 MBq) in good RCY (31 ± 5% decay corrected to start of synthesis), and concentrations of (4.32 ± 0.90) × 10^8^ microbubbles per mL, within in 60–70 min. Incorporation of the [^18^F]AlF-lipid into microbubble shells was confirmed by radio-HPLC-analysis of the centrifuge washing infranatant, which show a decreased percentage of the [^18^F]AlF-lipid compared to the [^18^F]AlF-Tz (Fig. S5, ESI†), inferring the incorporation of [^18^F]AlF-lipid into the MBs.

Successful ^18^F-labelling of MBs was also confirmed during the centrifugal purification process, by comparing the activity of the infranatant from successive centrifugal washes to that of the remaining microbubble foam layer (Fig. 4). Following the third wash, almost all remaining activity (>95%) resulted from the microbubbles. To prove that the radioactivity of the [^18^F]AlF-MBs was specific to [^18^F]AlF-lipid and not [^18^F]AlF-Tz, the microbubbles were dissolved in methanol and analysed by radio-HPLC. The resulting chromatogram showed only [^18^F]AlF-lipid, with no [^18^F]AlF-Tz detected (Fig. S6, ESI†).

**Fig. 4:**
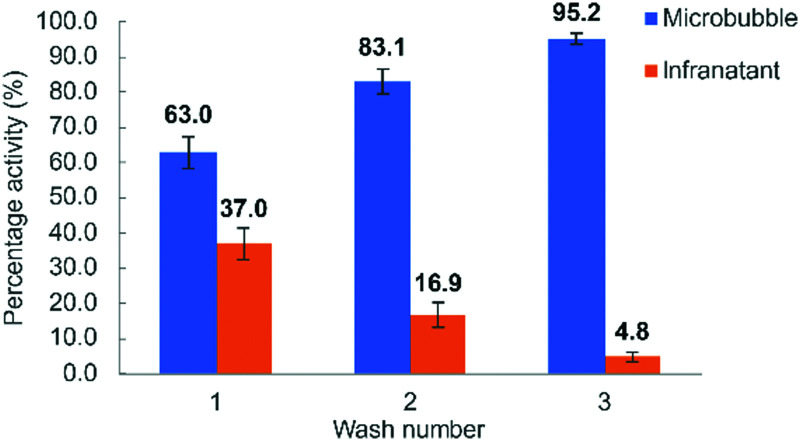
Comparison of activities of microbubble foam against infranatant after successive centrifuge washes. Reactions were performed in triplicate (*n* = 3), values presented as mean ± SD.

To minimise unnecessary exposure to radioactivity when synthesising [^18^F]AlF-Tz, the radiosynthesis was automated using the GE FASTLab^TM^ platform. An automated sequence was developed and [^18^F]AlF-Tz was produced from larger starting activities of [^18^F]fluoride (1067 ± 58 MBq) in 60–66% RCY within 45–50 min (details in ESI†). This was consistent with a reported automated synthesis of a similar [^18^F]AlF-tetrazine conjugate.^29^

Automation enabled the production of [^18^F]AlF-MBs in higher activities of 136 ± 6 MBq. The results of the [^18^F]AlF-MBs produced are summarised in Table 1.

**Table 1:** Comparison of ^18^F-labelled microbubble production using the manual, automated, and kit-based approaches. Reactions were performed in triplicate (*n* = 3), values presented as mean ± SD

Method	Starting activity (MBq)	End activity (MBq)	D.C. yield (%)	Synthesis time (min)
Manual	227 ± 25	48 ± 12	31 ± 5	60–70
Automated	1067 ± 58	136 ± 6	22 ± 1	85–90
Kit-based	65 ± 5	13 ± 2	30 ± 2	50–60

To support the potential clinical translation of this method, we developed a kit-based approach to producing the [^18^F]AlF-MBs. It was reasoned that a kit-based TCO-microbubble formulation could potentially be ^18^F-labelled in one pot (Fig. 5), owing to the fast reaction kinetics of the tetrazine–TCO IEDDA reaction.^31^ This would facilitate the development of new phospholipid-based MB formulations, including targeted MBs, since the TCO-functionalised lipid and biomolecule-lipid conjugate could be lyophilised with the other lipid components in the same vial prior to activation to form microbubbles, similar to existing commercial microbubble formulations.

**Fig. 5:**
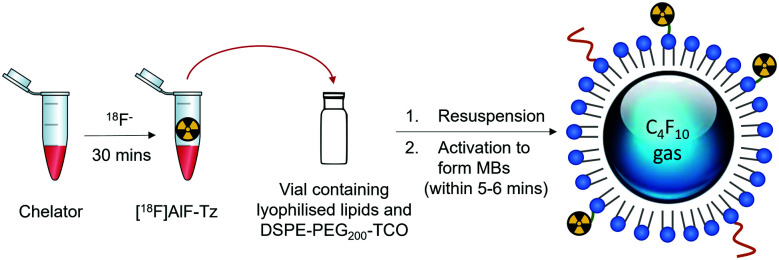
Development of a kit-based approach for ^18^F-labelling of microbubbles.

To investigate this, DSPE-PEG_200_-TCO was mixed and lyophilised in the same vial containing the rest of the lipids. Following resuspension of the lipids in a mixture of propylene glycol : glycerol : PBS (15 : 5 : 80), [^18^F]AlF-Tz in *ca.* 80 μL of EtOH was added. The vial was then sealed and purged with perfluorobutane, and agitated to form microbubbles by mechanical shaking for 1 min. To ensure sufficient time for the tetrazine–TCO ligation, the microbubble suspension was left to stand for a further 5 min before centrifugal purification.

The resulting microbubble suspension showed a 40–50% incorporation of radioactivity, consistent with the [^18^F]AlF-Tz TCO reaction yield. Analysis of the microbubble infranatant following centrifugal purification gave an identical profile to previous described experiments (Fig. 4), highlighting the feasibility of a kit-based TCO-lipid formulation for ^18^F radiolabelling of microbubbles.

To further facilitate the kit-based labelling procedure, such that the purification of [^18^F]AlF-Tz could be eliminated, the reaction co-solvent for ^18^F-labelling of the chelator was changed from MeCN to EtOH, a more good manufacturing process (GMP) compatible solvent (Fig. 1, step A). Interestingly, EtOH as the co-solvent resulted in a lower reaction yield (80%), compared to MeCN, which exhibited full (>95%) incorporation of [^18^F]AlF after 20 min. Nonetheless, extending the reaction time to 30 min resulted in >95% [^18^F]AlF chelation, thereby eliminating the need for a cartridge-based purification prior to incubation with the microbubbles. Using this kit-based approach, [^18^F]AlF-MBs could be produced in 30 ± 2% RCY, consistent to that of the previous methods, albeit with a lower activity of 13 ± 2 MBq. The decreased activity of the microbubbles is sufficient for multi-animal *in vivo* biodistribution studies, with currently reported studies requiring only 0.37–7.4 MBq for each injection.^12–14,16^ Since (1–5) × 10^7^ MBs are required per injection for pre-clinical US imaging, and this work generates microbubbles with activities of 0.3–3.2 MBq/10^7^ microbubbles, the requirements for both PET and US imaging are met, enabling the [^18^F]AlF-MBs to be used for both modalities in the same study.

In conclusion, we present the first method to generate [^18^F]AlF-labelled microbubbles. This approach offers a convenient method to generate radiolabelled microbubbles with higher activities, and hence larger dose, compared to previous attempts with ^68^Ga and ^18^F. The facile [^18^F]AlF-labelling procedures and efficient tetrazine–TCO IEDDA ‘click’ reaction also enable reliable and reproducible generation of the [^18^F]AlF-MBs. Using this robust method, we designed and developed the first kit-based approach for producing radiolabelled microbubbles with clinical translation in mind. With the continued development of new microbubble formulations bearing targeting vectors for molecular ultrasound imaging, this kit-based approach would enable easy customisation of new phospholipid-based formulations for early *in vivo* evaluation of their pharmacokinetics and biodistribution.

JHT acknowledges the Imperial College President's scholarship scheme for funding. EOA acknowledges funding support from Imperial College NIHR Biomedical Research Centre award (WSCC_P62585), Imperial College Experimental Cancer Medicines award C1312/A25149, and the Medical Research Council grant (MR/N020782/1). NJL thanks EPSRC Programme Grant ‘MITHRAS’ (EP/S032789/1) and the CDT in Medical Imaging (EP/L015226/1).

## Conflicts of interest

There are no conflicts of interest.

## Supplementary Material

CC-057-D1CC04790F-s001
