# Supplementary material for: A kit-based aluminium-[18F]fluoride approach to radiolabelled microbubbles
Source: Chem Commun (Camb). 2021 Oct 15;57(88):11677–80. doi: 10.1039/d1cc04790f (PMC8567295; doi:10.1039/d1cc04790f)
Supplement: CC-057-D1CC04790F-s001 [file CC-057-D1CC04790F-s001.pdf]

Electronic Supplementary Material (ESI) for ChemComm.
This journal is © The Royal Society of Chemistry 2021

Supplementary Data

Development of ^{18}F-labelled Microbubbles using [^{18}F]AlF Method and IEDDA Tetrazine-TCO 'click' Reaction

Jin Hui Teh,[a,b] Marta Braga,[b] Louis Allott,[b,c] Chris Barnes,[b] Javier Hernández-Gil,[a,b] Meng-Xing Tang,[d] Eric O. Aboagye,*[b] Nicholas J. Long*[a]

a. Department of Chemistry, Imperial College London, UK. Email: n.long@imperial.ac.uk.
b. Department of Surgery & Cancer, Imperial College London, UK. Email: eric.aboagye@imperial.ac.uk.
c. Positron Emission Tomography Research Centre, Faculty of Health Sciences, University of Hull, UK.
d. Department of Bioengineering, Imperial College London, UK.

Table of Contents

1. General Considerations
2. Organic Synthesis
3. Microbubble Production
4. Radiochemistry
5. Radio-HPLC and radio-TLC chromatograms
6. NMR spectra
7. Mass spectra

Experimental

1 General Considerations

Reagents: Anhydrous dichloromethane and acetonitrile were obtained under nitrogen from a PureSolv multiple dispensing solvent drying system and degassed for at least 30 minutes before use. Anhydrous DMF was purchased from Sigma Aldrich. Flash column chromatography was performed using Geduran® Si 60 Silicagel (Merck). 1,2-Dipalmitoyl-*sn*-glycero3-phosphocholine (DPPC, >99%), 1,2-Dipalmitoyl-*sn*-glycero-3-phosphate(sodium salt) (DPPA, >99%), 1,2-Distearoyl-*sn*-glycero-3-phosphoethanolamine (18:0 PE-NH2, >99%,), 1,2-Distearoyl-*sn*-glycero-3-phosphoethanolamine-N-[amino(polyethylene glycol)-2000] (ammonium salt) (DSPE-PEG(2000)-NH2, >99%) were purchased from Avanti Polar Lipids. All other reagents and solvents were used as purchased unless otherwise specified.

Instruments and characterisation: ^{1}H, ^{13}C and ^{19}F NMR were obtained using Bruker AV-400 spectrometers. Chemical shifts (δ) are reported in ppm relative to residual undeuterated solvent signals ($CDCl_3$ δH = 7.26 ppm, δC = 77.16 ppm; DMSO-d_6 δH = 2.50 ppm, δC = 39.52 ppm; CD_3CN: δH = 1.94 ppm, δC = 118.26 ppm ($CD_3\underline{C}N$); D_2O δH = 4.79 ppm; CD_3OD δH = 3.31 ppm, δC = 49.00 ppm). The following abbreviations are used to designate multiplicities: s = single, d = doublet, t = triplet, q = quartet, m = multiplet, quint = quintet, br = broad. Coupling constants (*J*) are reported in hertz (Hz). NMR spectra were analysed using MESTRELAB MestReNova software. High resolution mass spectra were recorded using a Waters LCT Premier (ESI) spectrometer by the Imperial College Department of Chemistry Mass Spectrometry service. Liquid Chromatography Mass Spectrometry (LC-MS) analyses were performed on a Waters LC-MS system: Waters 2767 autosampler for samples injection and collection; Waters 515

HPLC pump to deliver the mobile phase to the source; Waters 3100 mass spectrometer with ESI; and Waters 2998 Photodiode Array (detection at 200-600 nm), equipped with XBridge C_{18} reverse-phase columns with dimensions 4.6 mm × 100 mm. Reverse-phase purifications were carried out on an Isolera™ Spektra System using Biotage® SNAP Ultra C18 cartridges (12 g), and gradients are indicated throughout the text. Thin Layer Chromatography (TLC) was performed using TLC silica gel 60 F_{254} (aluminium sheets 20 × 20 cm for analytical runs and glass plates 20 × 20 cm for preparative TLC purifications of phospholipids. Phospholipids were visualised by charring with 5% primuline in acetone:water (8:2 *v/v*), with lipids appearing as yellow spots under 365 nm irradiation. Mass spectra (*m/z*) of phospholipids were run on a MALDI micro MX-TOF mass spectrometer from waters. Samples were spotted 1:1 *v/v* with a matrix solution (matrix indicated in text) and measured in linear positive mode.

Analytical HPLC chromatograms were obtained using an Agilent 1200 series instrument equipped with a flow-ram detector (Lablogic, Sheffield, UK), and integrated using Laura 6 software (Lablogic, Sheffield, UK). Semi-preparative HPLC was performed using a Waters HPLC. Analytical radio-HPLC chromatograms were obtained using an Agilent 1200 series instrument equipped with a flow-ram detector (Lablogic, Sheffield, UK). Radio-HPLC chromatograms were integrated using Laura 6 software (Lablogic, Sheffield, UK). Columns, flow rate, and mobile phases are indicated in the text.

The lipid-coated decafluorobutane-filled microbubbles were produced using a modified formulation (refer to section 4 for further details). 1 mL of the lipid solution was sealed in a 2 mL glass vial, and the headspace was purged with decafluorobutane at room temperature. The microbubbles were produced by mechanical agitation using a dental HL-AH High Speed Digital Amalgamator Amalgam Capsule Blend Mixer (4000 rpm for 30 s, two cycles).

2 Organic Synthesis

NODA-tetrazine conjugate:

N,*N*',*N*''-tritosyldiethylene triamine, *N*,*N*',*N*''-tritosyl-1,4,7-triazacyclononane and 1,4,7-triazacyclononane was synthesized according to the procedure reported by Lippard *et al.*[1] 1,4-bis(tert-butoxycarbonylmethyl)-1,4,7-triazanonane was synthesised according to the procedure reported by Shetty *et al.*[2]

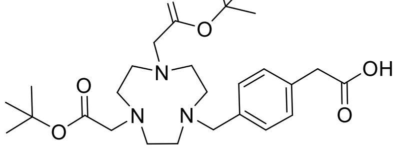

NO2A^{t}Bu-MPAA (2):[3] A suspension of 4-(bromomethyl)phenylacetic acid (0.62 mmol, 141.00 mg) in acetonitrile (4 mL) was added dropwise to a suspension of 1,4-bis(*tert*-butoxycarbonylmethyl)-1,4,7-triazacyclononane (0.56 mmol, 200.00 mg) and K_2CO_3 (1.12 mmol, 154.00 mg) in acetonitrile (8 mL). The resulting yellow suspension was stirred at 25 ^oC for 24 hours and monitored by TLC (CH_2Cl_2/MeOH; 9/1). Upon completion, the mixture was filtered, and the filtrate was concentrated *in vacuo*. The product

was purified by flash chromatography using an Isolera™ Spektra System (Biotage® SNAP Ultra C18 cartridge (12 g), A: H_2O with 0.1% TFA, B: CH_3CN with 0.1% TFA. Gradient: 0-100% B, product eluted at 25-30% CH_3CN), and the collected fractions were lyophilized to give a pale-yellow solid (110 mg, 39%).

^{1}H NMR (400 MHz, $CDCl_3$, *δ*): 8.22 (1H, br s), 7.49 (2H, d, $^{3}J_{HH}$ = 7.6 Hz), 7.34 (2H, d, $^{3}J_{HH}$ = 7.6 Hz), 4.36 (2H, s), 3.68 (2H, s), 3.55-2.81 (16H, m), 1.45 (18H, s); ^{13}C NMR (101 MHz, $CDCl_3$, *δ*): 174.4, 170.0, 136.6, 131.0, 130.6, 128.9, 82.9, 60.3, 57.0, 51.4, 50.4, 48.1, 40.8, 28.1; HRMS (ES^+, TOF): *m/z* $[M+H]^+$ calc. for $C_{27}H_{44}N_3O_6$ 506.3230, found 506.3222.

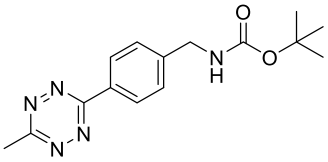

***Tert*-butyl (4-(6-methyl-1,2,4,5-tetrazin-3-yl)benzyl)carbamate:**[4] A high pressure reaction tube was treated with *tert*-butyl (4-cyanobenzyl)carbamate (2.00 mmol, 464 mg), CH_3CN (20.00 mmol, 1050 µL), nickel(II)trifluoromethanesulfonate (1.00 mmol, 356.00 mg) and hydrazine monohydrate (100.00 mmol, 6.2 mL). The tube was sealed and heated at 60 ^{o}C for 72 hours, following which sodium nitrite (40.00 mmol, 2.82 g) in H_2O (10 mL) was added dropwise to the mixture. 1 M HCl was then added dropwise until the pH reached 3 and gases stopped evolving, at which point the mixture had turned bright red. The product was extracted with EtOAc (3 x 40 mL), and the combined organic layers were washed with H_2O (3 x 20 mL), dried over $MgSO_4$, filtered and concentrated *in vacuo*. The crude pink solid was purified by flash column chromatography (CH_2Cl_2/Et_2O gradient 100:0 *v:v* to 96:4 *v:v*) to give a dark pink solid (261 mg, 43%).

^{1}H NMR (400 MHz, $CDCl_3$, *δ*): 8.55 (2H, d, $^{3}J_{HH}$ = 8.3 Hz), 7.50 (2H, d, $^{3}J_{HH}$ = 8.3 Hz), 4.99 (1H, br s), 4.43 (2H, d, $^{3}J_{HH}$ = 6.1 Hz), 3.09 (3H, s), 1.48 (9H, s); ^{13}C NMR (101 MHz, $CDCl_3$, *δ*): 167.2, 163.9, 155.9, 144.0, 130.8, 128.2, 128.1, 79.8, 44.4, 28.4, 21.2. HRMS (APCI, TOF): *m/z* $[M]^+$ calc. for $C_{15}H_{19}N_5O_2$ 301.1533, found 301.1533.

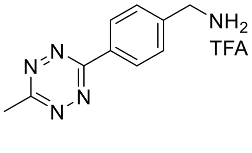

(4-(6-Methyl-1,2,4,5-tetrazin-3-yl)benzylamine (1):[4] *Tert*-butyl (4-(6-methyl-1,2,4,5-tetrazin-3-yl)benzyl)carbamate (0.15 mmol, 45.00 mg) was treated with TFA/CH_2Cl_2 (1:1,*v:v*, 4 mL) and stirred for 2 h at room temperature. The solvent was removed *in vacuo* to give the TFA salt of 4-(6-methyl-1,2,4,5-tetrazin-3-yl)benzylamine as a pink solid (41.70 mg, 88% yield).

^{1}H NMR (400 MHz, CD_3OD, *δ*): 8.63 (2H, d, $^{3}J_{HH}$=8.4 Hz), 7.72 (2H, d, $^{3}J_{HH}$ = 8.4 Hz), 4.26 (2H, s), 3.06 (3H, s); ^{13}C NMR (101 MHz, CD_3OD, *δ*): 167.7, 163.5, 137.4, 133.0, 129.4, 128.0, 42.5, 19.7); ^{19}F NMR (377 MHz, CD_3OD, *δ*): -73.5; HRMS (APCI, TOF): *m/z* $[M+H]^+$ calc. for $C_{10}H_{12}N_5$ 202.1087, found 202.1096.

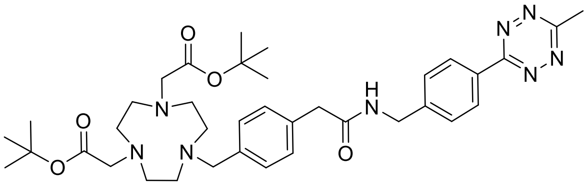

NO2A^{t}Bu-MPAA-tetrazine (3): Under a nitrogen atmosphere, NO2A^{t}Bu-MPAA **(1)** (0.12 mmol, 60.00 mg) and HBTU (0.14 mmol, 53.7 mg) were dissolved in anhydrous DMF (1 mL). Triethylamine (0.12 mmol, 16.50 µL) was added and the pale yellow suspension was stirred for 15 mins at 40 ^oC. (4-(6-Methyl-1,2,4,5-tetrazin-3-yl)benzylamine **(2)** (0.12 mmol, 23.70 mg) in anhydrous CH_2Cl_2 (1 mL) and triethylamine (0.16 mmol, 23.50 µL) were added, and the resulting pink solution was stirred at 40 ^oC for 24 hours. Completion of the reaction was monitored by TLC (90:10 CH_2Cl_2:MeOH). The resulting solution was concentrated *in vacuo* to give a pink solid, which was purified by flash column chromatography (CH_2Cl_2/MeOH gradient 100:0 *v:v* to 80:20 *v:v*), and washed with Et_2O to give a dark pink solid (50.05 mg, 60%).

^1H NMR (400 MHz, $CDCl_3$, δ): 8.46 (2H, d, $^3J_{HH}$ = 8.4 Hz), 7.53 (2H, d, $^3J_{HH}$ = 8.1 Hz), 7.44 (2H, d, $^3J_{HH}$ = 8.4 Hz), 7.37 (2H, d, $^3J_{HH}$ = 8.1 Hz), 6.36 (1H, t, $^3J_{HH}$ = 6.1 Hz), 4.50 (2H, d, $^3J_{HH}$ = 6.1 Hz), 4.40 (2H, s), 3.65 (2H, s), 3.33-3.17 (8H, m), 3.07 (3H, s), 3.07-3.00 (2H, m), 2.80-2.59 (6H, m), 1.45 (18H, s); ^{13}C NMR (101 MHz, $CDCl_3$, δ): 170.7, 170.5, 167.2, 163.9, 143,4, 137.1, 131.2, 130.7, 130.4, 129.5, 128.4, 128.1, 82.3, 65.8,z 59.4, 56.7, 51.6, 50.4, 47.4, 43.5, 43.3, 28.1, 21.2, 15.3; HRMS (ES$^+$, TOF): *m/z* $[M+H]^+$ calc. for $C_{37}H_{53}N_8O_5$ 689.4139, found 689.4163.

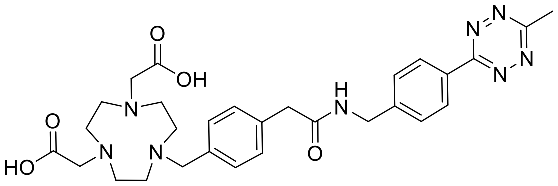

NODA-MPAA-tetrazine (4): NO2A^{t}Bu-MPAA-tetrazine **(3)** (0.017 mmol, 12.30 mg) was treated with TFA/CH_2Cl_2 (1:1,*v:v*, 2 mL) and stirred for 5 h at room temperature. The solvent was removed *in vacuo,* and the crude product was purified by flash chromatography using an Isolera™ Spektra System (Biotage® SNAP Ultra C18 cartridge (12 g), A: H_2O with 0.1% TFA, B: CH_3CN with 0.1% TFA. Gradient: 0-100% B) The fractions were lyopholized to give the desired product as a pink solid (10.50 mg, 99%).

^1H NMR (400 MHz, CD_3OD, δ): 8.50 (2H, d, $^3J_{HH}$ = 8.4 Hz), 7.61 (2H, d, $^3J_{HH}$ = 8.1 Hz), 7.51 (2H, d, $^3J_{HH}$ = 8.4 Hz), 7.46 (2H, d, $^3J_{HH}$ = 8.1 Hz), 4.51 (2H, s), 4.44 (2H, s), 3.67 (2H, s), 3.54-3.40 (2H, m), 3.27-3.11 (8H, m), 3.05 (3H, s), 2.86-2.64 (6H, m); ^{13}C NMR (101 MHz, CD_3OD, δ): 172.8, 168.5, 164.7, 144.6, 138.6, 132.2, 131.8, 131.3, 130.7, 129.2, 128.7, 59.4, 55.5, 51.8, 50.4, 47.3, 43.8, 43.1, 21.4; HRMS (ES$^+$, TOF): *m/z* $[M+H]^+$ calc. for $C_{29}H_{37}N_8O_5$ 577.2880, found 577.2877.

Phospholipid synthesis:

DSPE-PEG$_{200}$-TCO was synthesised as described by Hernandez *et al*.:[4]

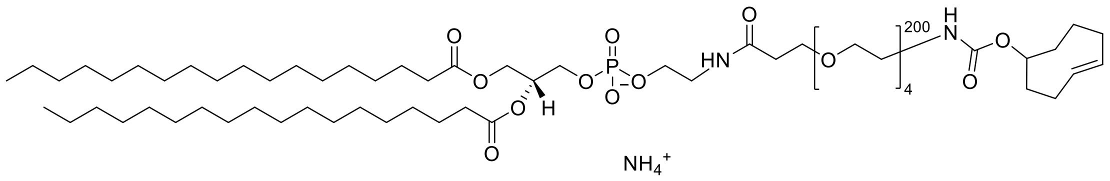

DSPE-PEG$_{200}$-TCO: 18:0 PE-NH$_2$ (10 mg, 13.3 µmol) was dissolved in a mixture of anhydrous $CHCl_3$:MeOH (0.56:0.44 *v:v*) and triethylamine (50 µL). A solution of TCO-PEG$_4$-NHS (10 mg, 19.43 µL in 0.3 mL $CHCl_3$) was added, and the solution was stirred for 5 h. The solvent was removed *in vacuo*, and the product was purified by prep TLC ($CHCl_3$: MeOH: H_2O 8:2:0.2 *v:v:v*) to give an off-white solid (7.2 mg, 48%).

^{1}H NMR (400 MHz, CD_3OD, δ): 5.64–5.52 (1H, m), 5.48–5.37 (1H, m), 5.23 (1H, m), 4.41–4.35 (1H, m), 4.30–4.23 (1H, m), 4.21–4.14 (1H, m,), 4.00 (4H, m), 3.84–3.38 (18H, m), 3.02 (2H, m), 2.52–2.41 (2H, m), 2.40-2.33 (2H, m), 2.30–2.25 (4H, m), 2.04-1.70 (12H, m), 1.64–1.55 (6H, m), 1.26 (58H, m), 0.88 (6H, t, $^3J_{HH}$ = 6.5 Hz); MALDI (matrix: CHCA); *m/z* $[M + 2Na]^+$ calc. for $[C_{62}H_{121}N_3O_{16}PN]^+$: 1190.7, found: 1190.6.

3 Microbubble Production

The lipid-coated, decafluorobutane-filled microbubbles (MBs) were produced using a modified formulation.[4] Hernandez *et al*. prepared MBs containing 1,2-dipalmitoyl-*sn*-glycero-3-phosphocholine (DPPC), 10 mol% PE-PEG$_4$-TCO, and 5 mol% 1,2-dipalmitoyl-*sn*-glycero-3-phosphoethanolamine-*N* [methoxy(polyethylene glycol)-2000] (DSPE-PEG$_{2000}$-NH$_2$). For this reason, we prepared MBs having 75 mol% DPPC, 10 mol% DPPA, 10 mol% PE-PEG$_4$-TCO, and 5 mol% DSPE-PEG$_{2000}$-NH$_2$. The total lipid concentration was between 0.85-0.88 mg/mL. The mixture of lipids was first dissolved in chloroform, dried over nitrogen gas, and then dried overnight *in vacuo* to remove residual solvent. Then, propylene glycol (150 µL), phosphate-buffered saline (PBS) (800 µL), and glycerol (50 µL) were added in order. This created a lipid suspension, which was stirred for 10 mins at room temperature, then left to stand for 10 mins. The vials were then sealed in a 2 mL glass vial, and the headspace purged with decafluorobutane. The microbubbles were produced *via* mechanical agitation to yield a cloudy solution.

To remove residual lipids, vesicles, and unreacted [^{18}F]AlF-NODA-tetrazine, the vial containing the microbubbles was put upside down and centrifuged at 400 *g* for 2 mins using a ROTINA 35R centrifuge. The microbubbles were collected as a concentrated cake on the top of the vial, and the infranatant was discarded. The microbubble cake was re-dispersed in 1 mL of PBS:glycerol 80:20 *v:v* and stored in the same vial with PFB headspace for further use.

Microbubble characterisation: Microbubble sizing and concentration were obtained using a bright-field microscope (Nikon Eclipse 50i, 40x objective) according to a protocol in our group.[5] Zeta potentials were recorded using a 1:100 diluted sample in PBS using a Malvern Nano ZetaSizer.

	TCO-modified
Concentration (MB/mL)	4.32×10^8
Mean diameter (µm)	1.98 ± 1.55
Range (µm)	0.50 – 9.97
Zeta potential (mV)	+8

4 Radiochemistry

Prior to usage, $^{18}F^-$ was trapped on a Sep-PAK Accell Plus QMA light cartridge (Cl^- form, Waters) and eluted with 0.9% *w:v* NaCl solution.

Radiosynthesis of [^{18}F]AlF-Tz

A mixture of NODA-MPAA-Tetrazine **(4)** in MeCN (12.5 µL, 100 nmol), 2 mM $AlCl_3$ in 0.5 M sodium acetate at pH 4.2 (50 µL, 100 nmol), purified [^{18}F]fluoride (100-250 MBq, 150 µL) and MeCN (200 µL) was incubated at 100 °C for 20 min. Upon completion, the reaction was then diluted in 0.1% TFA water (20 mL), trapped on an Oasis® HLB (30 mg) Light cartridge, washed with 0.1% TFA water (5 mL), and eluted with ethanol (300 µL). Analytical RP-HPLC (0.1% TFA H_2O:MeCN 95:5 *v/v* to 5:95 *v/v* gradient, phenomenex, Gemini 5u C18 110 A column, 1 mL/min flow rate): R_t [^{18}F]AlF-Tz = 8 min 48 sec.

Optimisation data for radiosynthesis of [^{18}F]AlF-NODA-MPAA-tetrazine

Entry	Organic co-solvent	Reaction Time (min)	Radiochemical Yield (%)[a]
1	EtOH	15	75
2	MeOH	15	73
3	MeCN	15	90
4	DMSO	15	88
5	DMF	15	88
6	MeCN	10	80
7	MeCN	15	93
8	MeCN	20	>95
9	MeCN	25	>95

Table S1: Optimisation of radiolabelling conditions for NODA-MPAA-tetrazine**.** [a]Calculated based on radio-HPLC

Entry	Aq:Org ratio	Time (min)	RCY(%)
1	3:2	15	81
2	1:1	15	82
3	2:3	15	87
4	2:3	20	>95

Table S2: Optimisation of ratio of aqueous to organic solvent.

Precursor Amount (nmol)	RCY (%)[a]
20	69
50	86
75	92
100	93

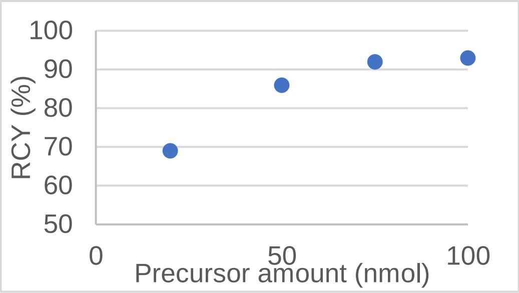

Table S3: Investigation of radiolabelling efficiency with respect to precursor amount. [a] calculated based on radio-HPLC.

Tetrazine-TCO reaction

To purified [^{18}F]AlF-Tz in ethanol (300 µL), DSPE-PEG$_{200}$-TCO (100 nmol) in ethanol (10 µL) was added. The reaction was incubated at 60 ^{o}C unsealed, allowing ethanol to evaporate. Completion of the reaction was monitored by radio-TLC (2 M NH_4OAc:MeOH 1:1 *v/v* – lipid remains at baseline, [^{18}F]AlF-Tz moves with solvent front). Radio-HPLC (0.1% SDS in 200 mM NH_4OAc:MeOH 50:50 *v/v* to 5:95 *v/v* gradient, phenomenex Aeris^{TM} 3.6 µm WIDEPORE C4 200 Å 250 x 4.6 mm column, 1 mL/min flow rate).

Automation:

A standard FASTlab synthesis manifold was used with silicone tubing. Purification cartridges were conditioned with EtOH (5 mL), water (10 mL) and dried with air (10 mL) prior to use.

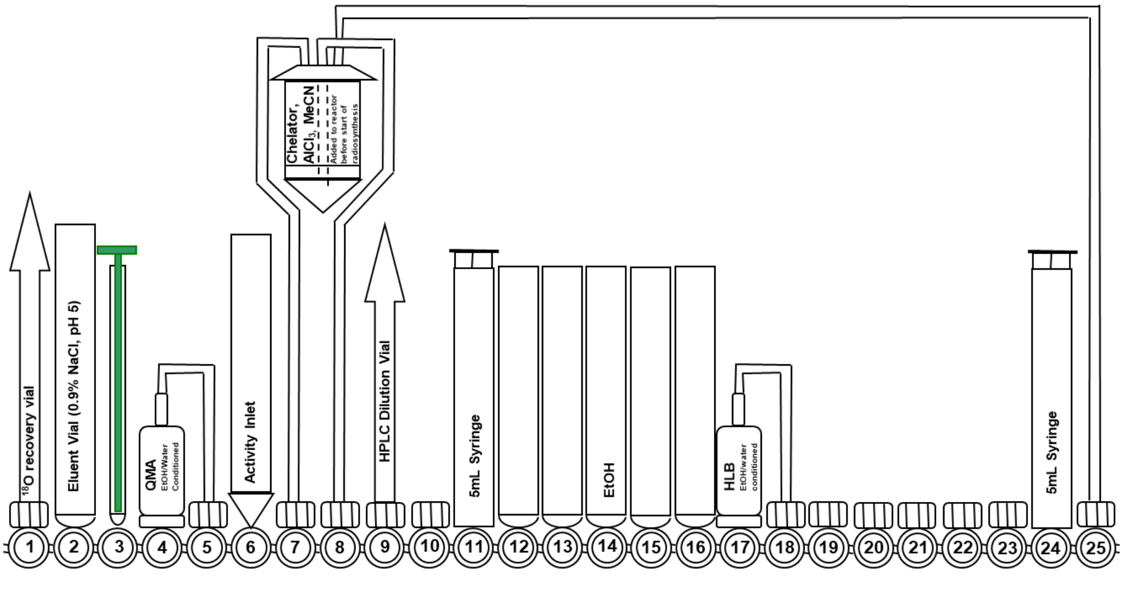

Cassette position	
1	^{18}O water recovery vial
2	Eluent vial (1000 µL 0.9% saline)
3	1 mL syringe
4/5	QMA cartridge
6	Activity inlet
7/8	Reaction vessel
9	Dilution vial: water (25 mL)
10	HPLC loop
11	5 mL syringe
12	Empty
13	Empty
14	5 mL ethanol
15-16	Empty
17/18	Oasis® HLB (30 mg) Light cartridge
19-23	Empty
24	5 mL syringe
25	Reaction vessel (vac line)

Table S4: FASTlab™ cassette setup

Automated radiosynthesis:

No-carrier-added aqueous [^{18}F]fluoride in enriched ^{18}O water is then delivered to the FASTlab™ radiosynthesis module and trapped onto a Waters Accell™ Plus QMA cartridge (Cl^- form, WAT023525) using the syringe at position 1. The [^{18}F]fluoride was eluted in 0.9% saline (pH 5, 1 mL) into the reaction vessel pre-loaded with ligand (25 µL, 8 mM in MeCN), $AlCl_3$ (100 µL, 2 mM in pH 4.2 NaOAc buffer), and MeCN (1 mL) at position 7/8. The reaction vessel was then heated at 100 ^{o}C for 20 mins, where nitrogen is bubbled through the reaction solution. After which, the reaction solution was diluted in 0.1% TFA H_2O (25 mL) at position 9, then passed through an Oasis® HLB (30 mg) Light cartridge. The product was eluted with ethanol (0.5 mL), giving 450-550 MBq of activity in 60-66% RCY.

Precursor amount (nmol)	Starting activity (MBq)	Isolated Activity (MBq)	Non-decay corrected RCY (%)	Decay-corrected RCY (%)
200	1100	500	45	60
	990	470	47	63
	1100	550	50	66

<u>[^{18}F]AlF-microbubbles (2-step production)</u>

[^{18}F]AlF-Tz in ethanol (300 µL) was added to a vial containing PE-PEG$_4$-TCO (0.13 mg, 0.11 µmol). The reaction vial was heated at 60 °C for 20 mins. After that, the volume of the reaction was reduced to approximately 100 µL, and this was added to a solution containing the rest of the lipids (DPPC 0.553 mg, DPPA 0.053 mg, DSPE-PEG$_{2000}$-NH$_2$ 0.1222 mg) dissolved in 1 mL of a solution of propylene glycol, glycerol, and phosphate-buffered saline (PBS) (15:5:80 *v/v/v*). The vial was sealed and purged with perfluorobutane, and the microbubbles were produced by mechanical agitation (5000 rpm for 30 s, two cycles). The microbubble suspension was then centrifuged for purification.

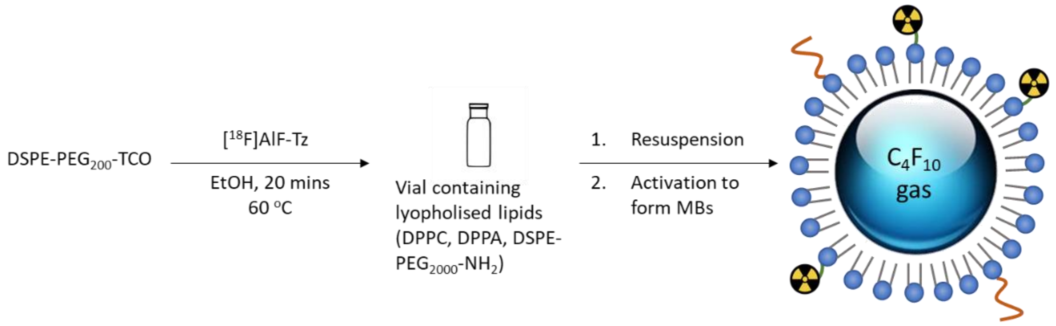

Figure S1: Two-step approach for development of ^{18}F-microbubbles

<u>[^{18}F]AlF-microbubbles (kit-based production)</u>

[^{18}F]AlF-Tz in ethanol/reaction solution (80 µL) was added to a vial containing PE-PEG$_4$-TCO (0.13 mg, 0.11 µmol) and the rest of the lipids (DPPC - 0.553 mg, DPPA - 0.053 mg, DSPE-PEG$_{2000}$-NH$_2$ - 0.1222 mg) dissolved in 1 mL of a solution of propylene glycol, glycerol, and phosphate-buffered saline (PBS) (15:5:80 *v:v:v*). The vial was sealed and purged with perfluorobutane, and the microbubbles were produced by mechanical agitation (5000 rpm for 30 s, two cycles). The microbubble suspension was left to stand for 5 mins, and then centrifuged for purification.

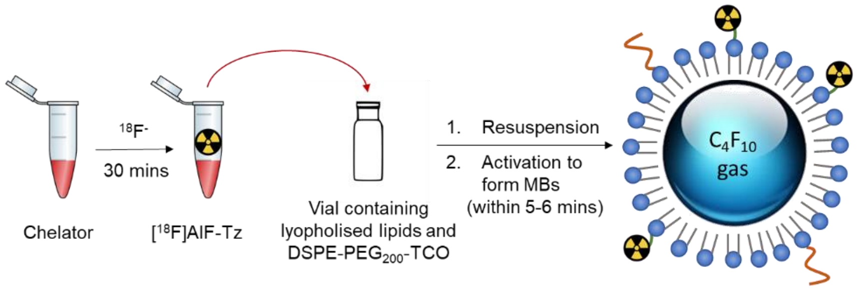

Figure S2: Kit-based approach for development of ^{18}F-microbubbles

5 Radio-HPLC and radio-TLC chromatograms

[^{18}F]AlF-Tz

Conditions: 0.1% TFA H_2O:MeCN 95:5 *v/v* to 5:95 *v/v* gradient, phenomenex, Gemini 5u C18 110 A column, 1 mL/min flow rate

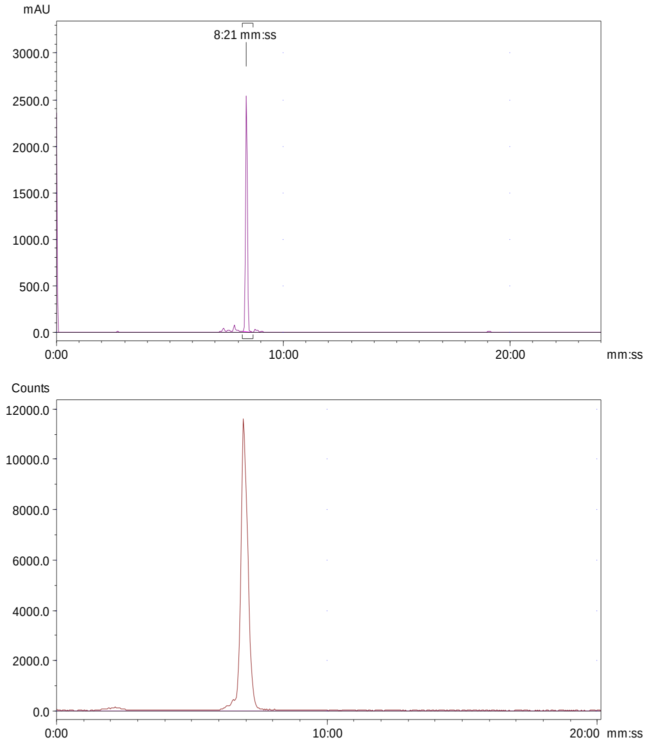

Figure S3: Top: HPLC chromatogram of NODA-MPAA-tetrazine. Bottom: radio-HPLC chromatogram of [^{18}F]AlF-tetrazine

Conditions: 0.1% SDS in 200 mM NH_4OAc:MeOH 50:50 *v/v* to 5:95 *v/v* gradient, phenomenex, Aeris™ 3.6 µm WIDEPORE C4 200 Å 250 x 4.6 mm column, 1 mL/min flow rate

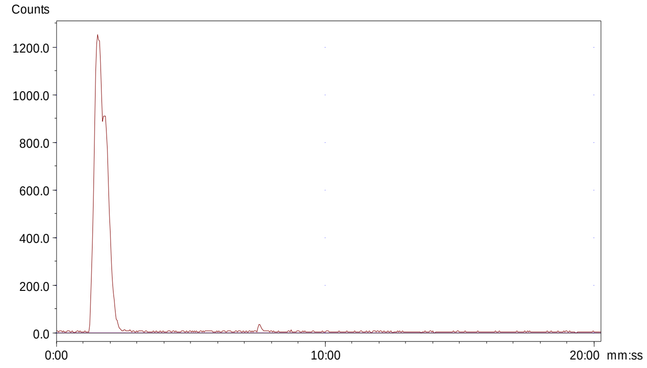

Stability

Table S5: HPLC chromatograms showing decomposition of [^{18}F]AlF-Tz, where a secondary peak is observed to form with a slightly shorter retention time compared to the main product peak

Time	Decomposition	Radio-HPLC chromatogram
1 h	7%	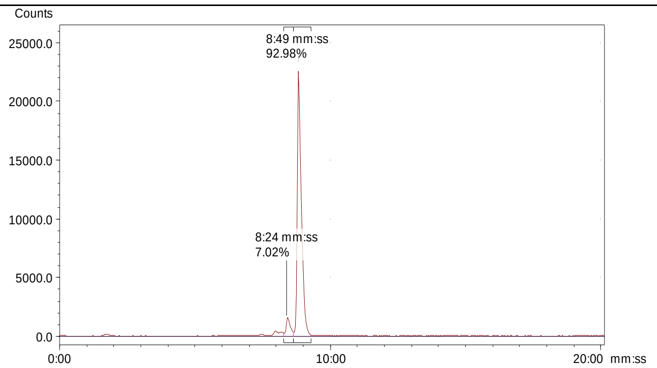
2 h	9%	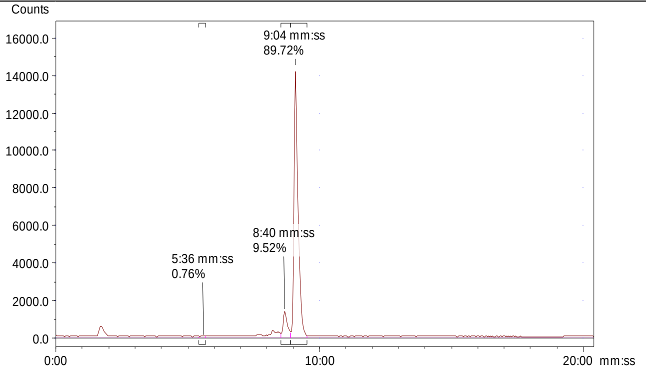
3 h	11%	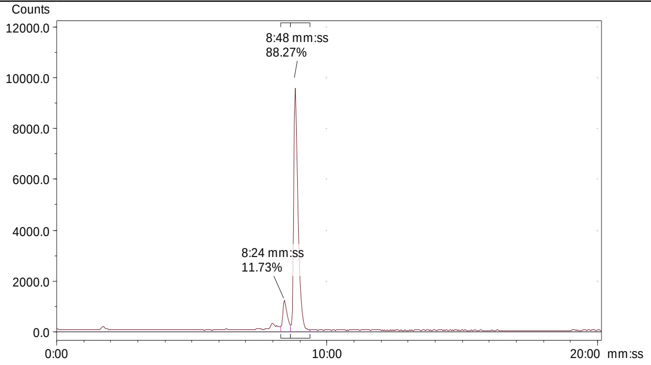
4 h	13%	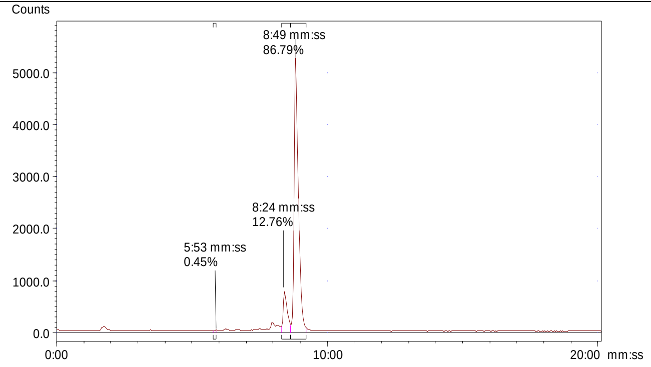

[^{18}F]AlF-Tz-TCO reaction to form [^{18}F]AlF-lipid

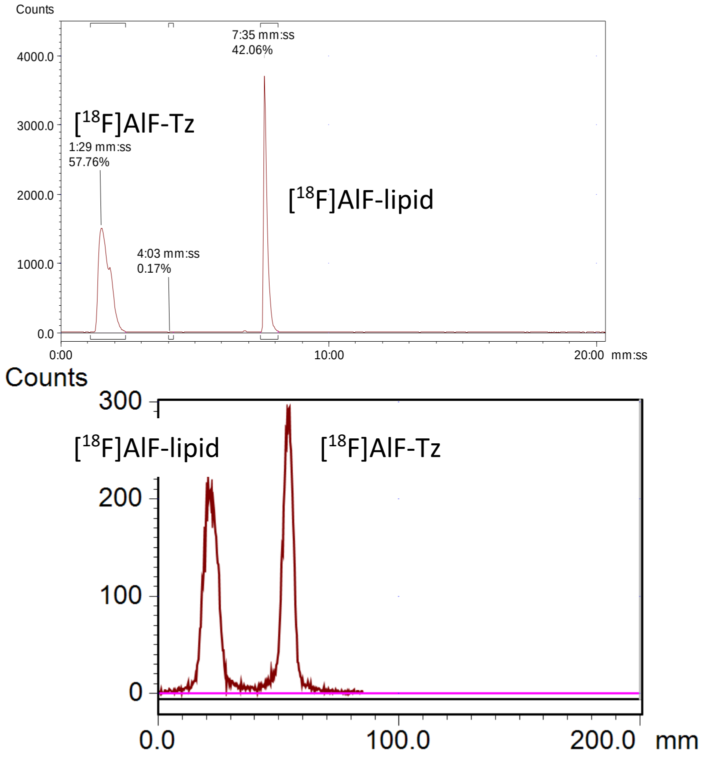

Figure S4: Top: radio-HPLC chromatogram of [^{18}F]AlF-Tz reaction with TCO. Bottom: Radio-TLC chromatogram of the same reaction.

[^{18}F]AlF-microbubbles

Before purification	After purification
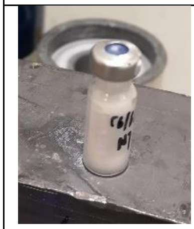	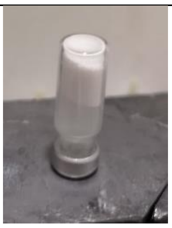

Picture of microbubble suspension before and after purification, where microbubbles are collected as a concentrated layer of foam at the top of the vial following centrifugation.

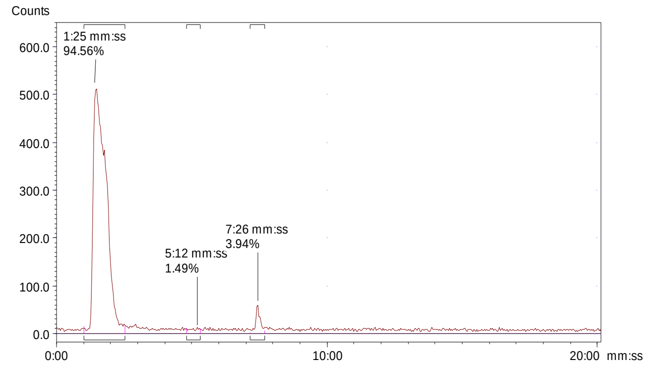

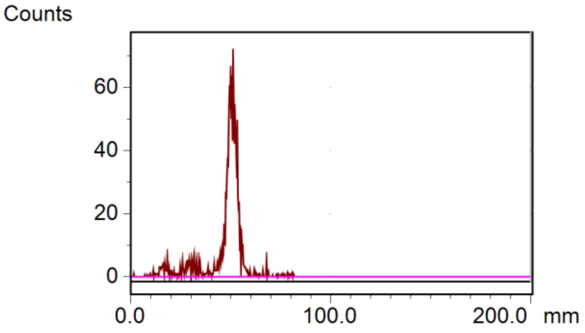

Figure S5: Top: radio-HPLC chromatogram of [^{18}F]AlF-microbubbles infranatant after centrifugation. Bottom: Radio-TLC chromatogram of infranatant. Both show decreased amount of [^{18}F]AlF-lipid after incorporation into microbubbles

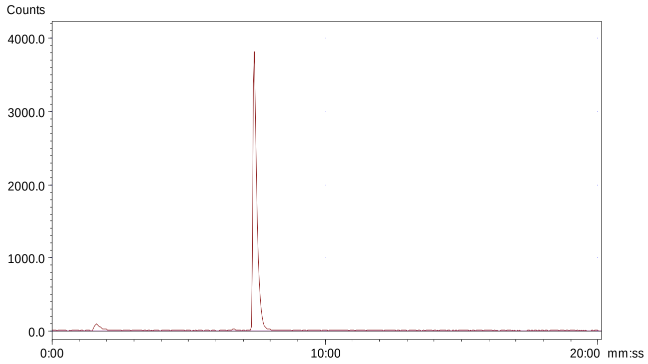

Figure S6: Radio-HPLC chromatogram of [^{18}F]AlF-microbubbles dissolved in MeOH after purification, only showing [^{18}F]AlF-lipid.

6 NMR Spectra

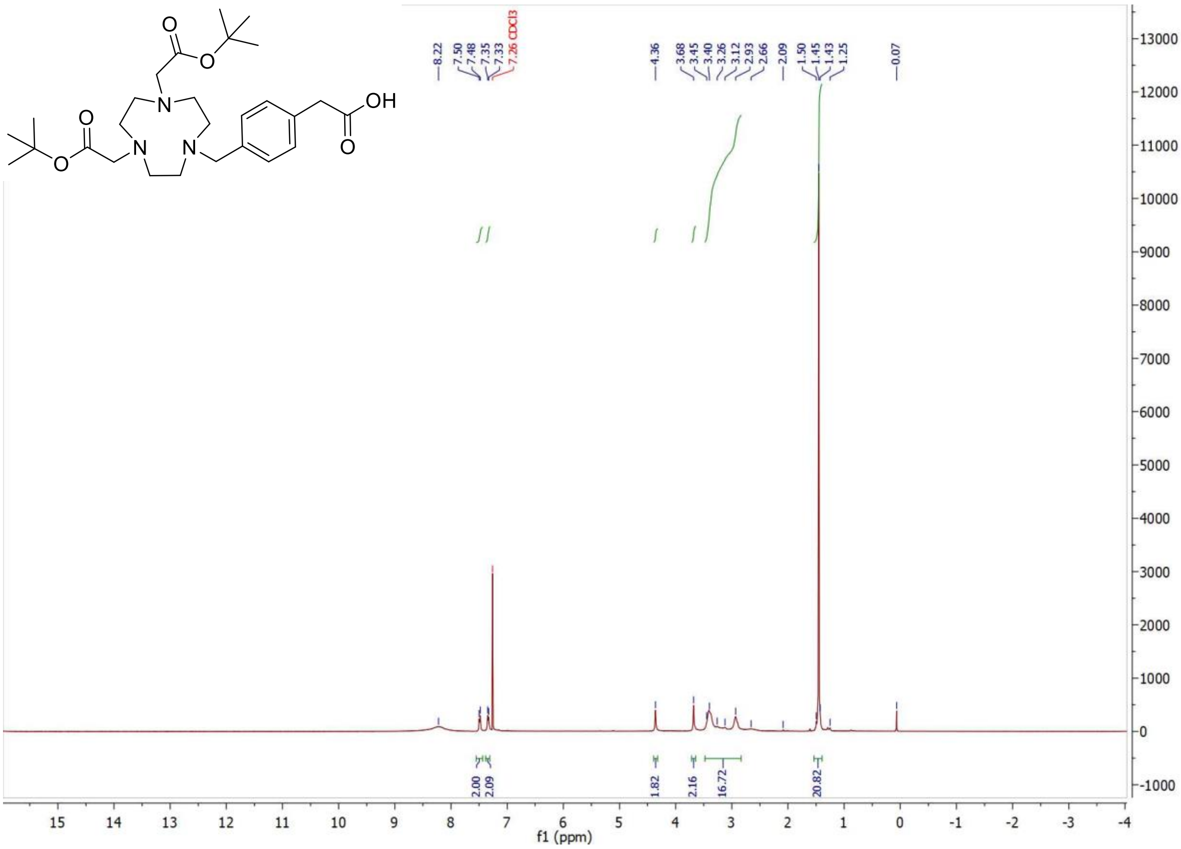

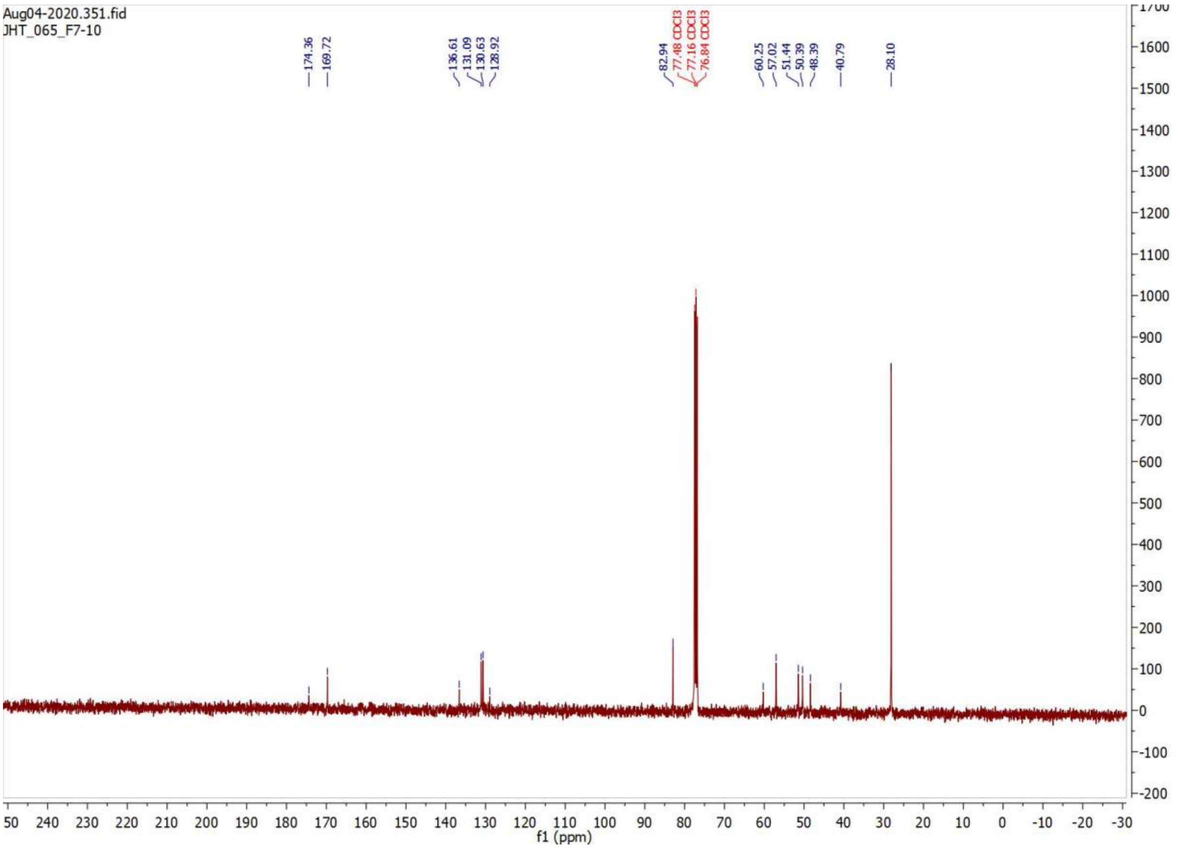

^{1}H and ^{13}C NMR spectra of tBu-NODA-MPAA **(2)** in $CDCl_3$

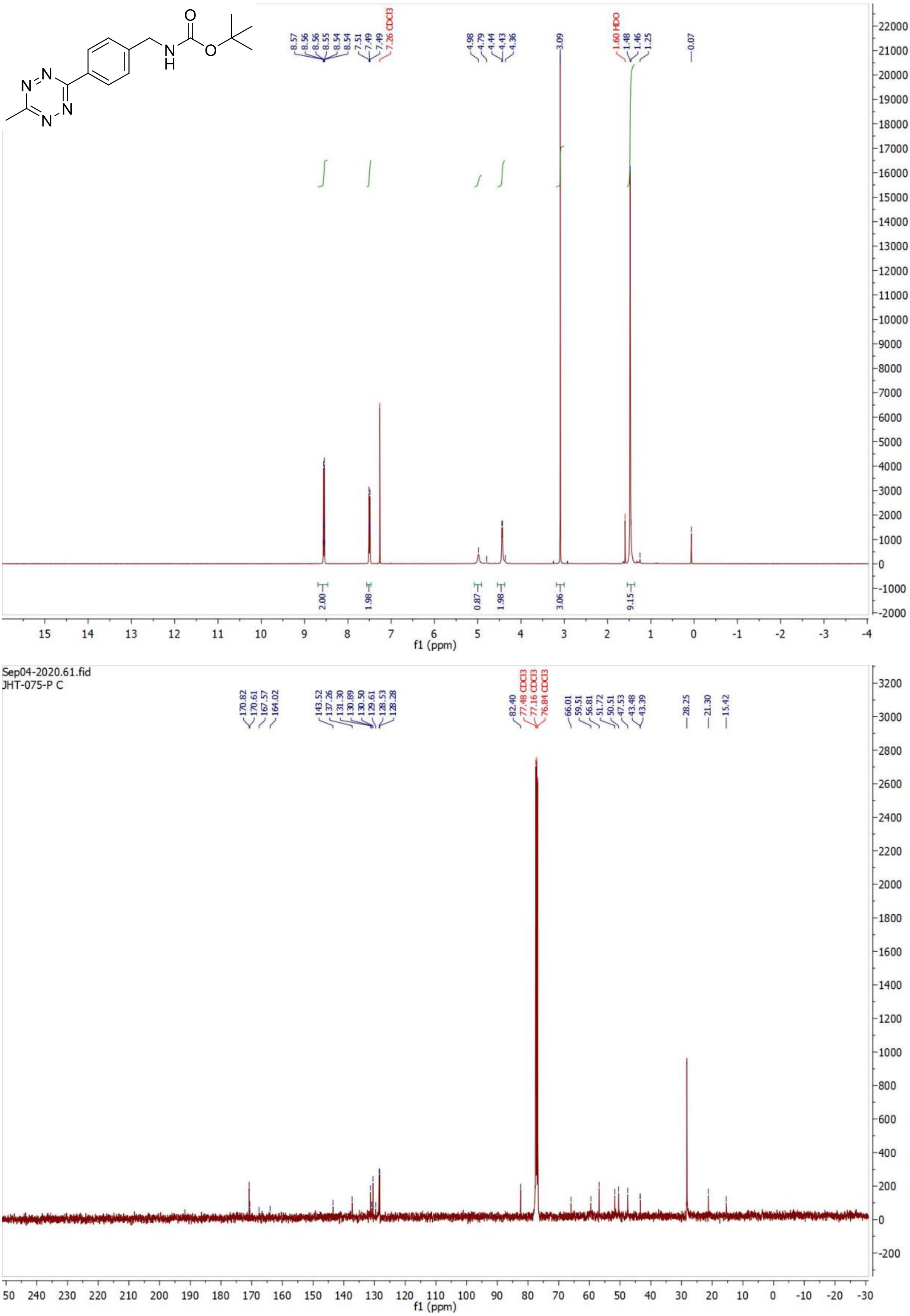

<u>^{1}H and ^{13}C NMR spectra of *tert*-butyl (4-(6-methyl-1,2,4,5-tetrazin-3-yl)benzyl)carbamate in $CDCl_3$</u>

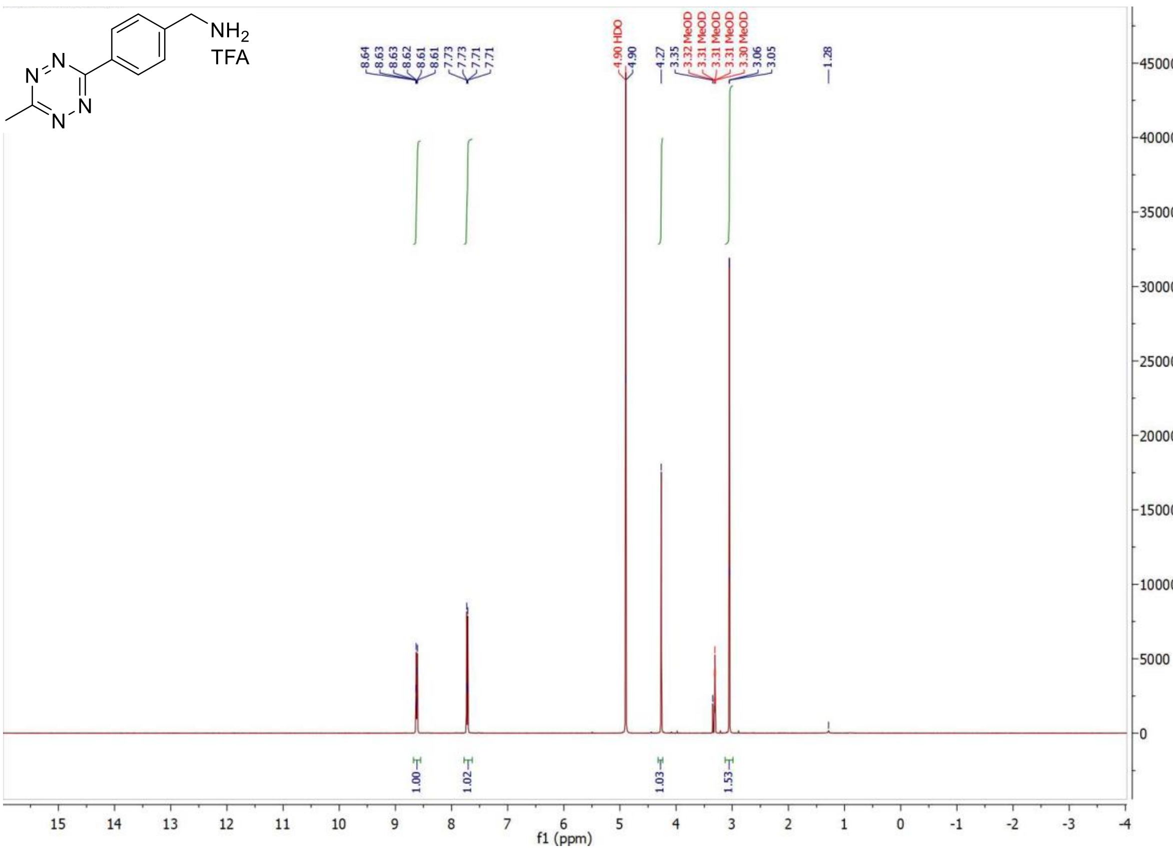

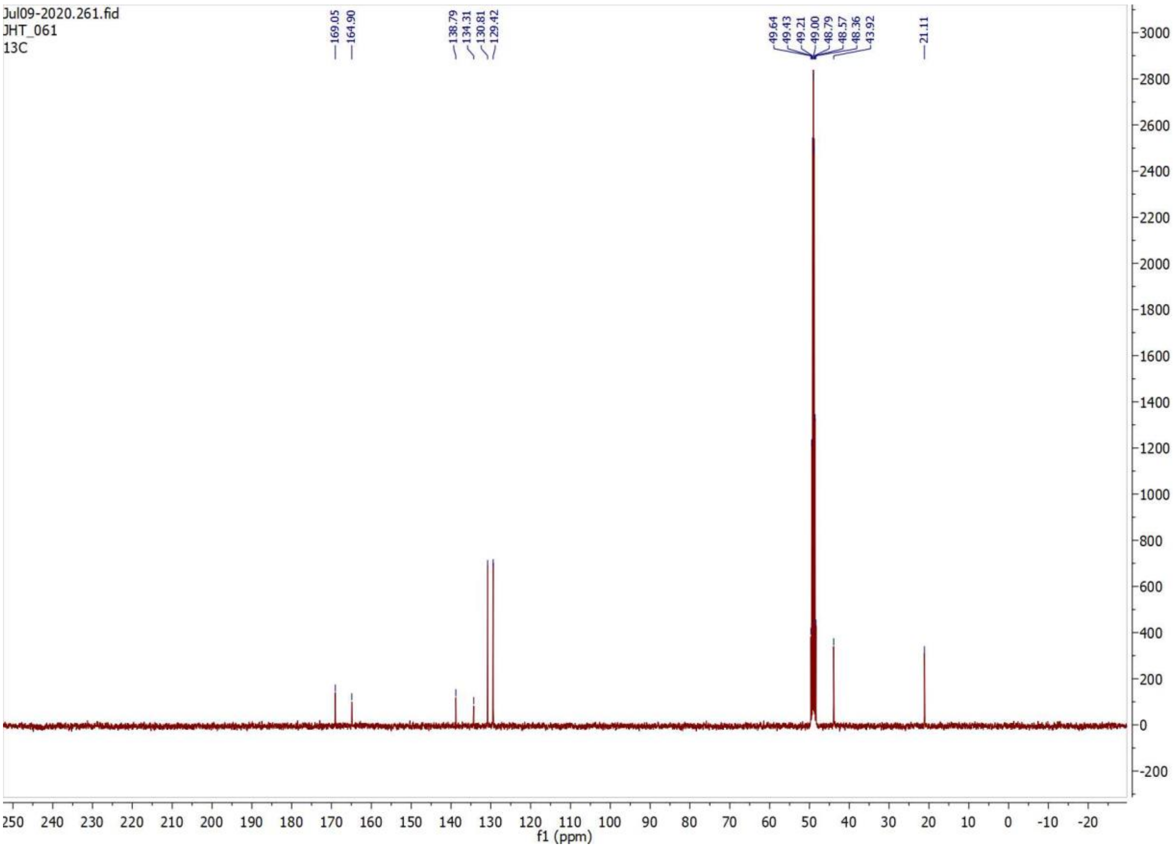

^{1}H and ^{13}C NMR spectra of (4-(6-methyl-1,2,4,5-tetrazin-3-yl)benzylamine **(1)** in *d*$_3$-MeOD

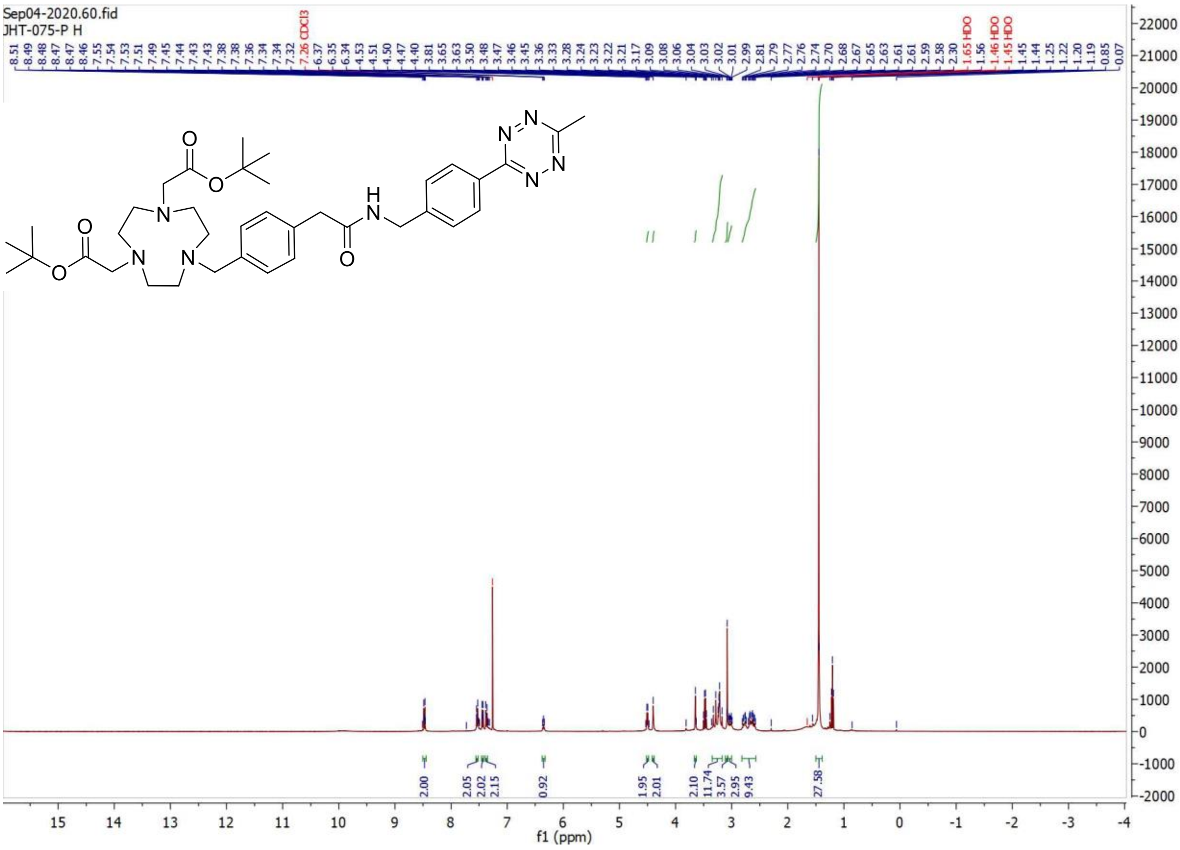

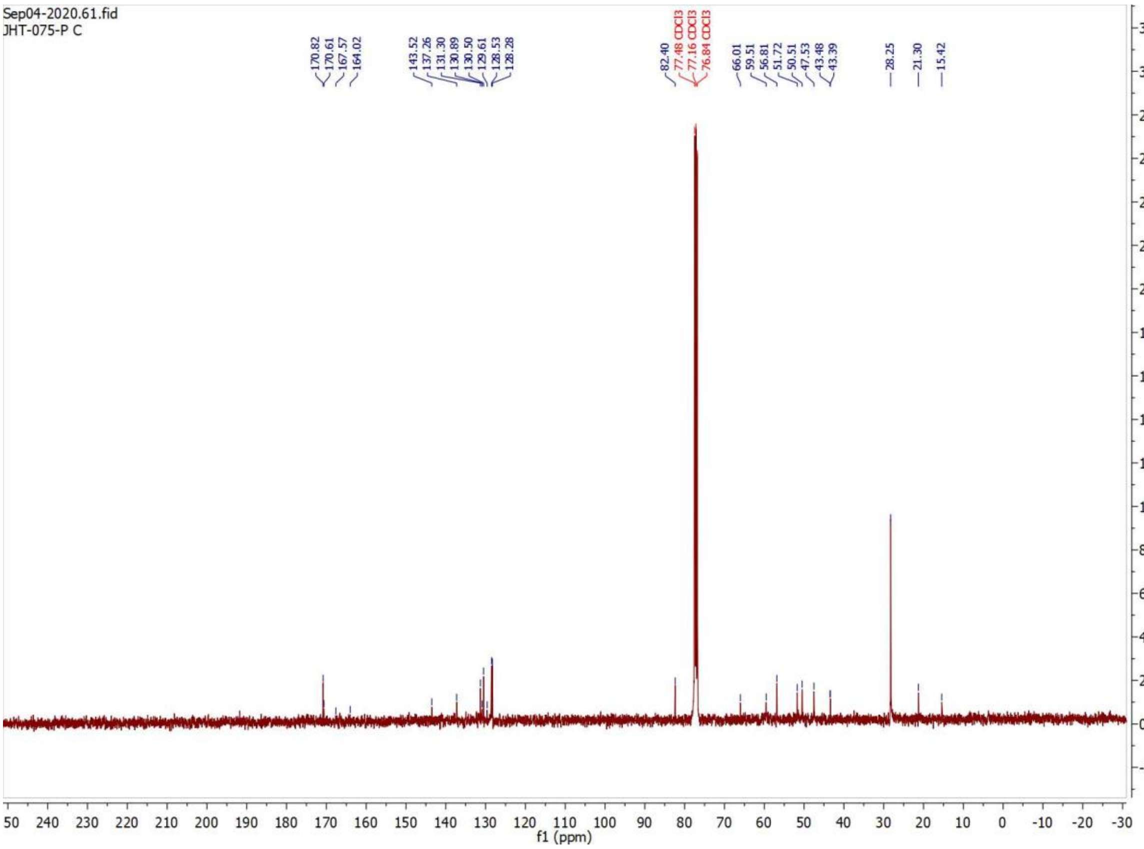

<u>^{1}H and ^{13}C NMR spectra of NO2A*t*Bu-MPAA-tetrazine **(3)** in $CDCl_3$</u>

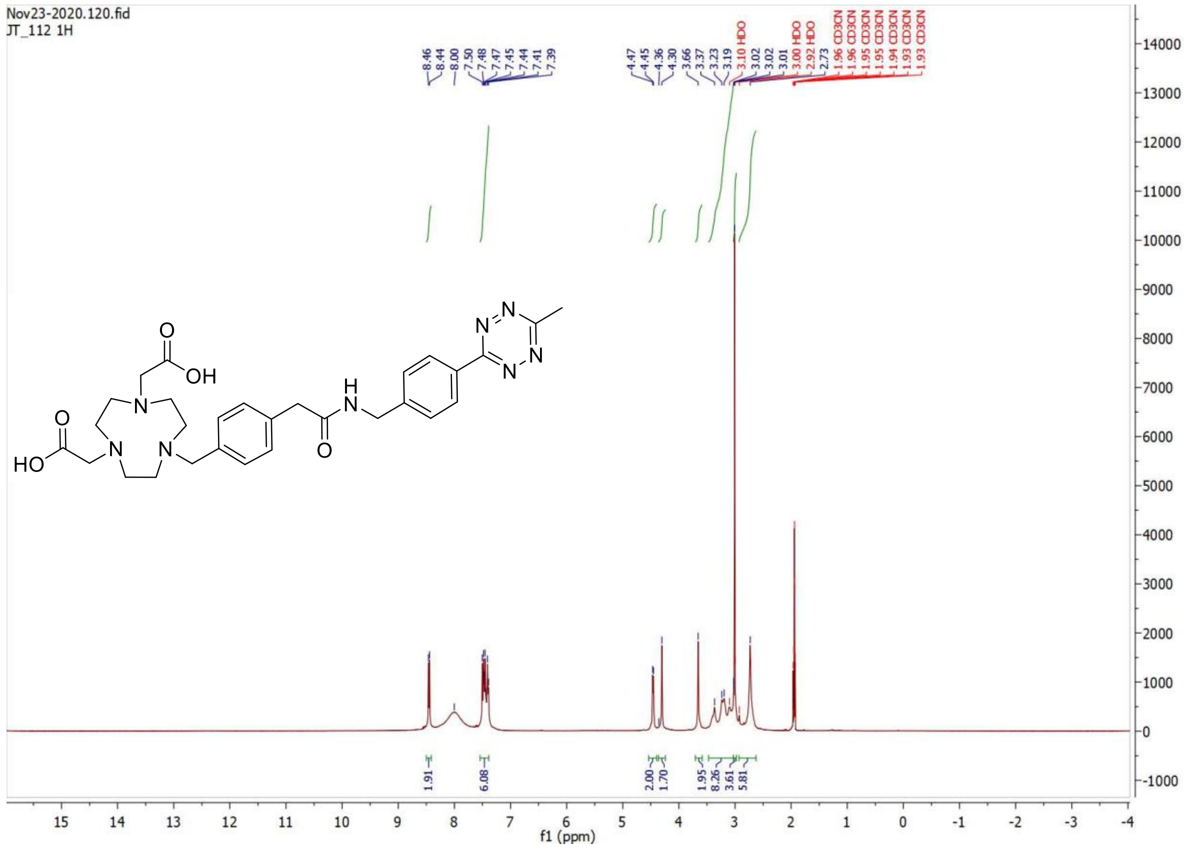

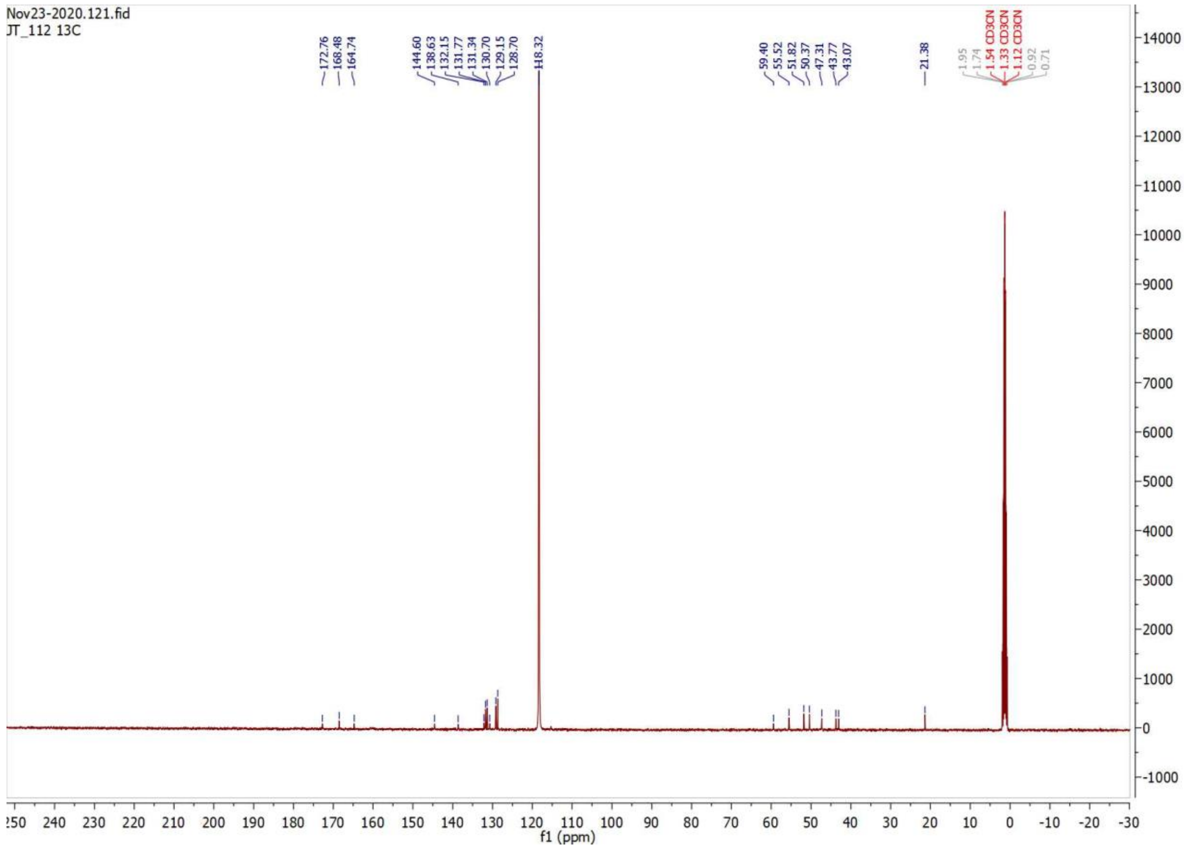

^{1}H and ^{13}C NMR spectra of NODA-MPAA-tetrazine **(4)** in CD_3CN

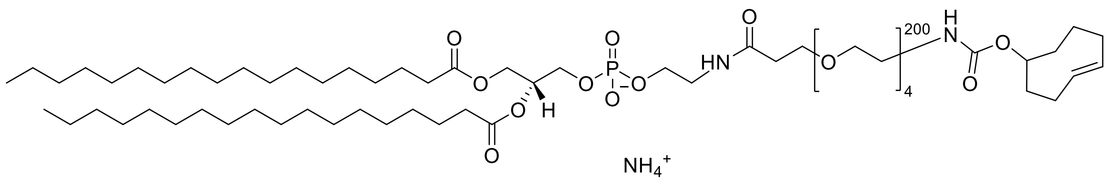

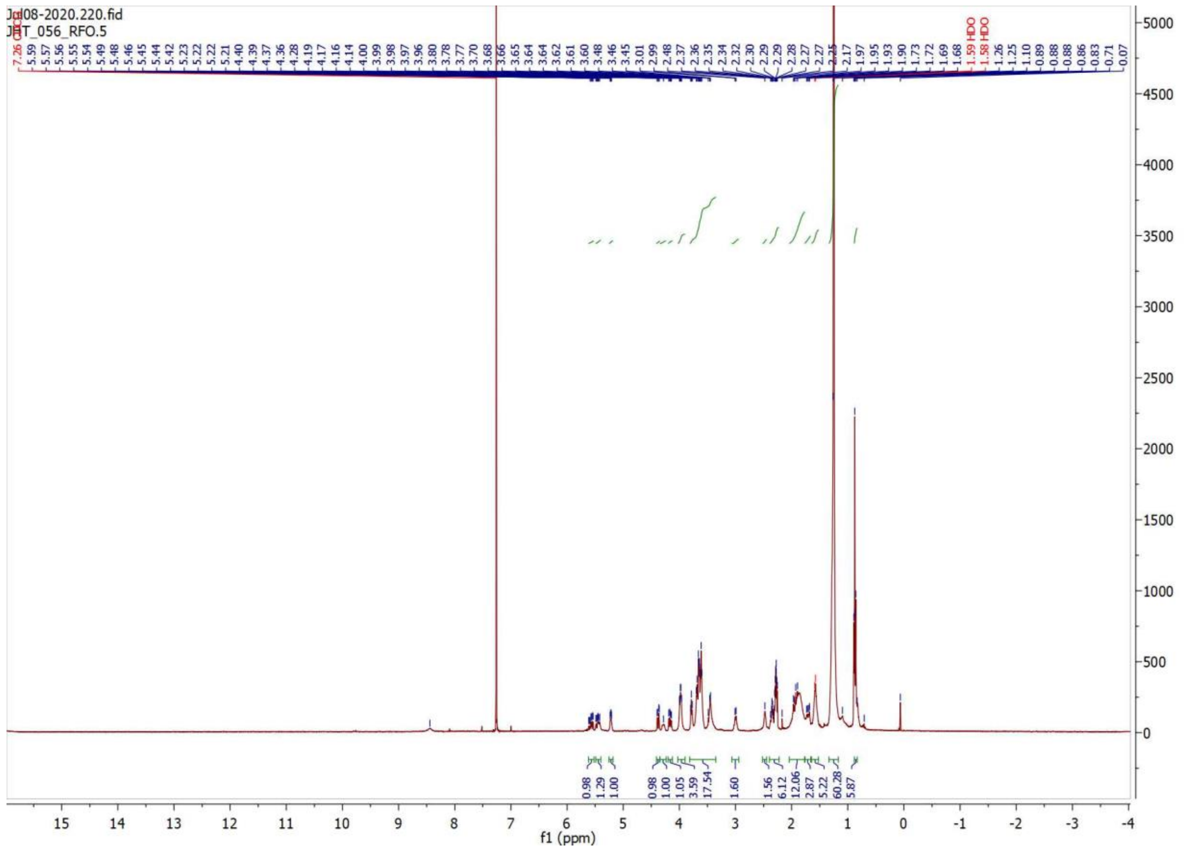

^{1}H NMR spectra of DSPE-PEG$_{200}$-TCO in $CDCl_3$

7 Mass Spectra

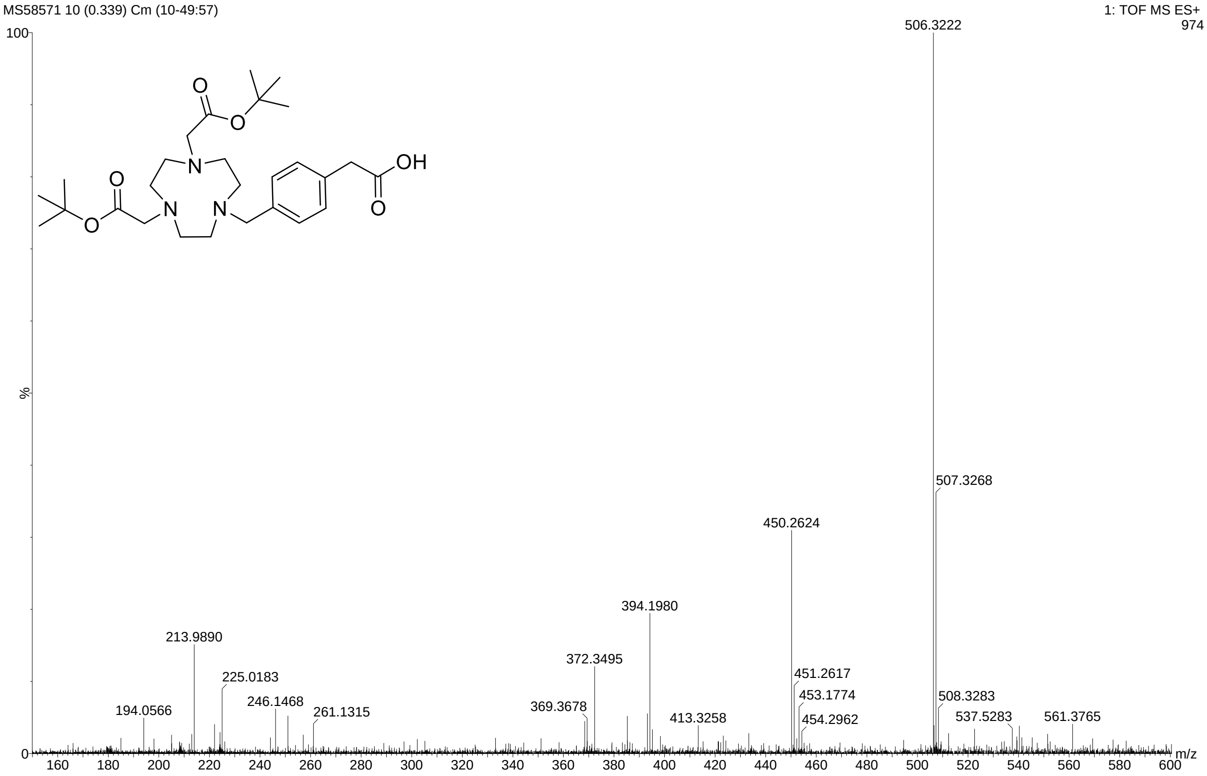

Mass spectrum of tBu-NODA-MPAA **(2)** [M+H]+ calc. for $C_{27}H_{44}N_3O_6$ 506.3230, found 506.3222

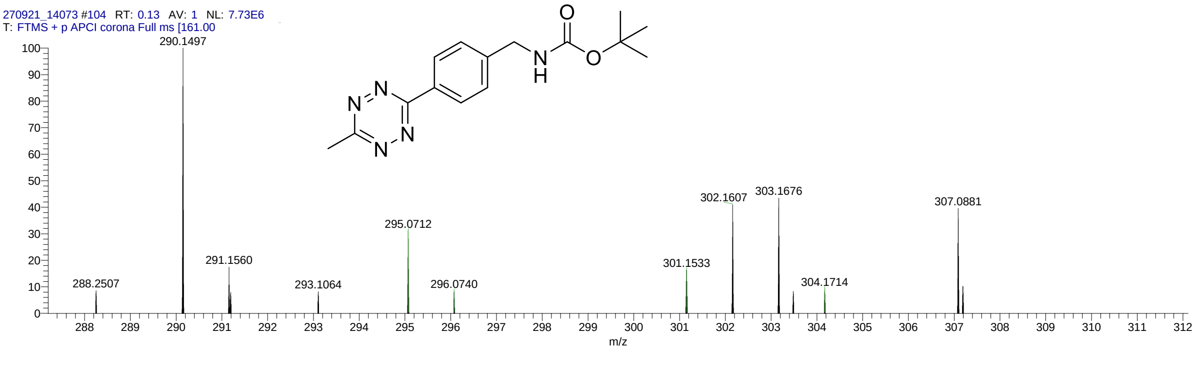

Mass spectrum of *Tert*-butyl (4-(6-methyl-1,2,4,5-tetrazin-3-yl)benzyl)carbamate *m/z* [M]+ calc. for $C_{15}H_{19}N_5O_2$ 301.1533, found 301.1533.

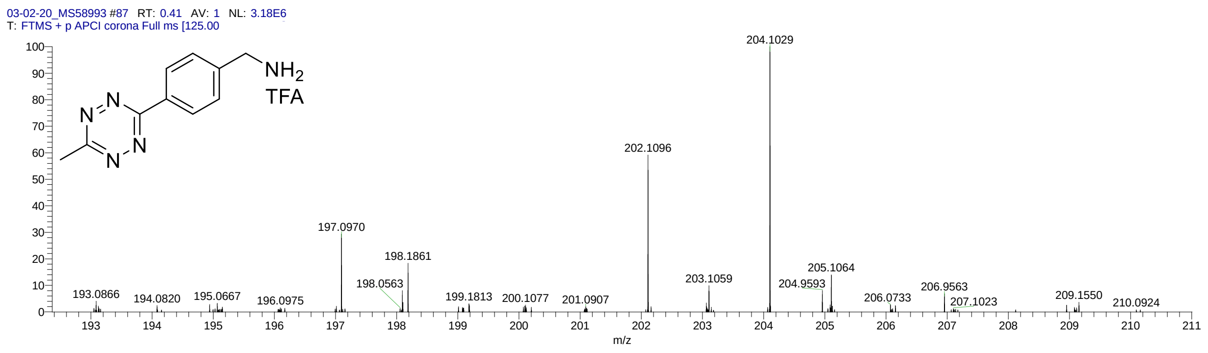

Mass spectrum of (4-(6-methyl-1,2,4,5-tetrazin-3-yl)benzylamine **(1)** *m/z* [M+H]+ calc. for $C_{10}H_{12}N_5$ 202.1087, found 202.1096.

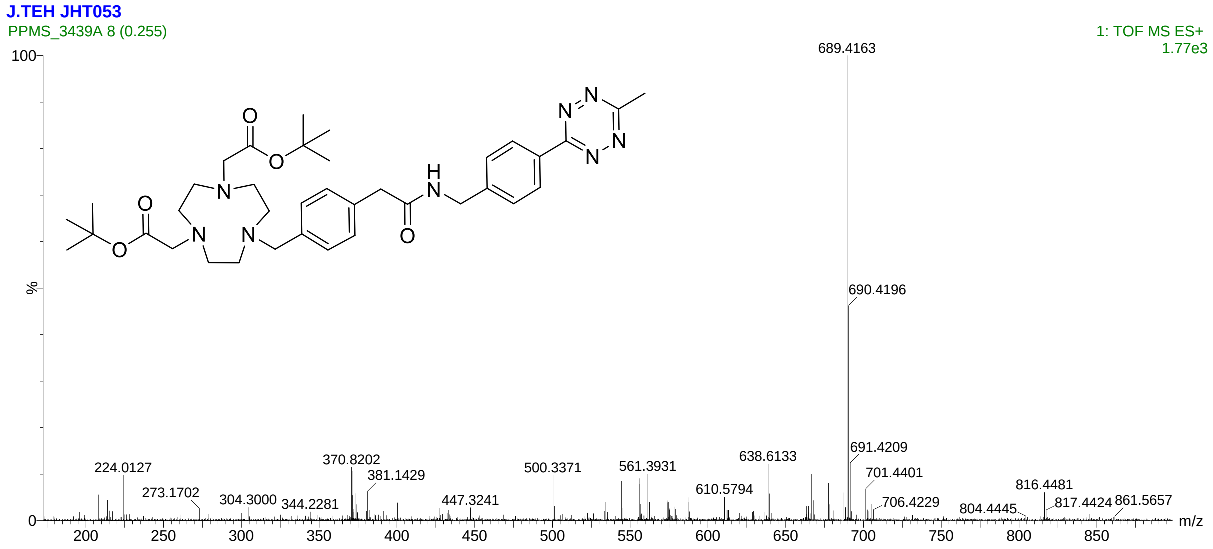

Mass spectrum of NO2A^{t}Bu-MPAA-tetrazine **(3)** *m/z* [M+H]+ calc. for $C_{37}H_{53}N_8O_5$ 689.4139, found 689.4163

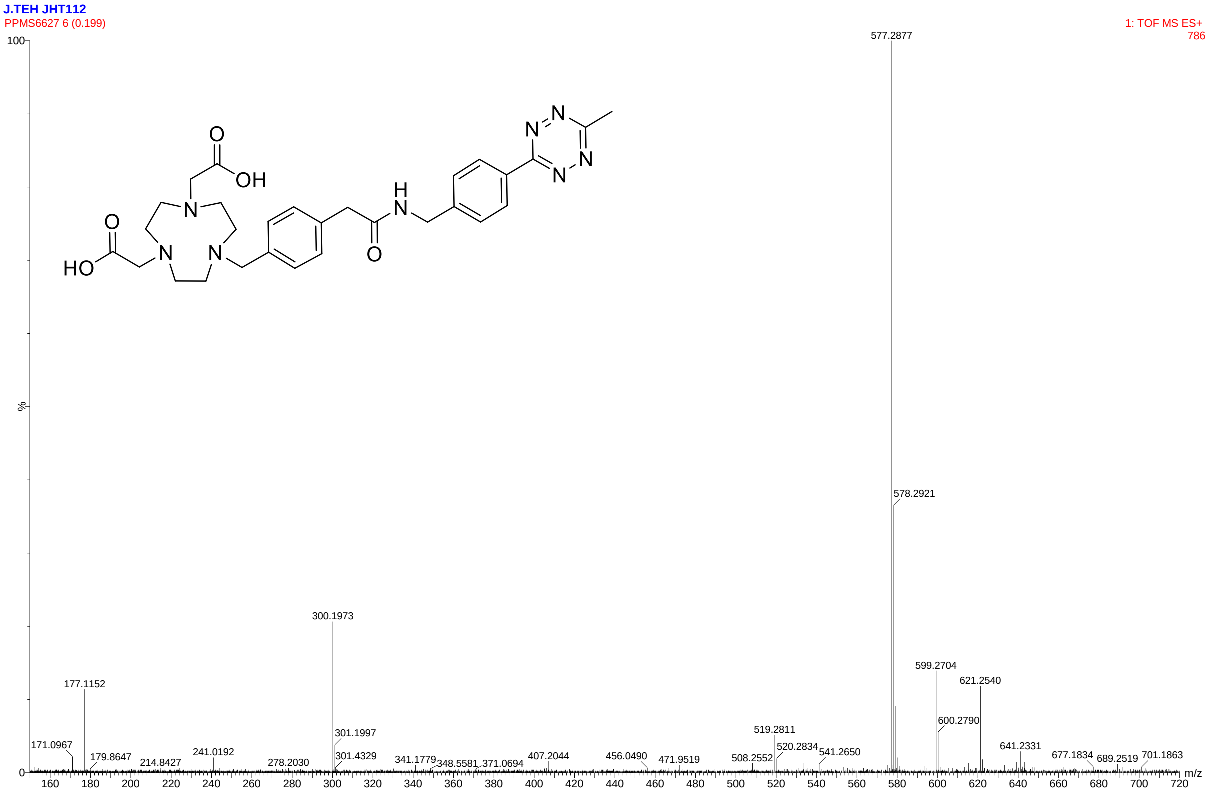

Mass spectrum of NODA-MPAA-tetrazine **(4)** *m/z* [M+H]+ calc. for $C_{29}H_{37}N_8O_5$ 577.2880, found 577.2877

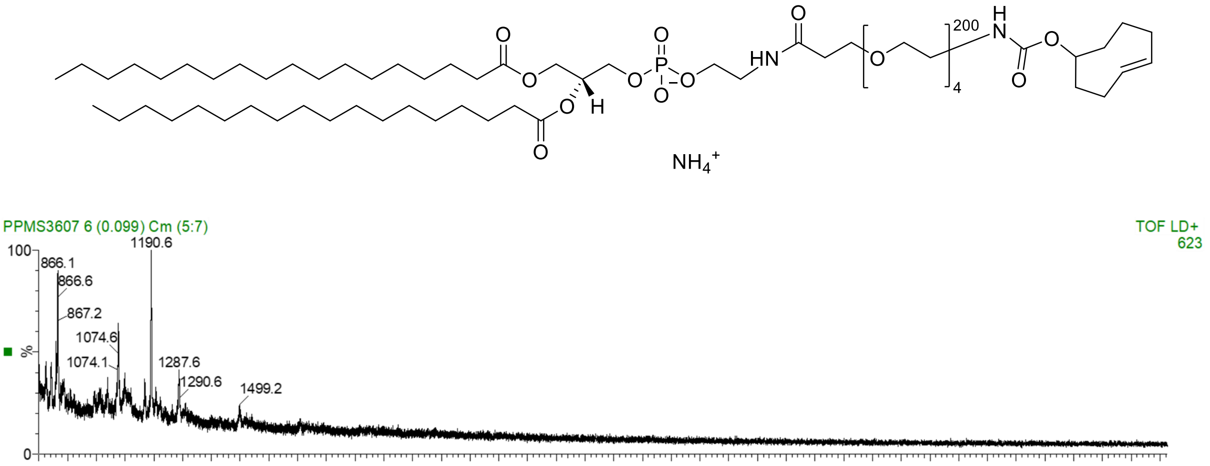

Mass spectrum of DSPE-PEG200-TCO *m/z* [M + 2Na]+ calc. for $[C_{62}H_{121}N_3O_{16}PN]^+$: 1190.7, found: 1190.6.

References:

1 R. Cao, P. Müller and S. J. Lippard, *J. Am. Chem. Soc.*, 2010, **132**, 17366–17369.

2 D. Shetty, S. Y. Choi, J. M. Jeong, J. Y. Lee, L. Hoigebazar, Y. S. Lee, D. S. Lee, J. K. Chung, M. C. Lee and Y. K. Chung, *Chem. Commun.*, 2011, **47**, 9732–9734.

3 C. A. D'Souza, W. J. McBride, R. M. Sharkey, L. J. Todaro and D. M. Goldenberg, *Bioconjug. Chem.*, 2011, **22**, 1793–1803.

4 J. Hernández-Gil, M. Braga, B. I. Harriss, L. S. Carroll, C. H. Leow, M.-X. Tang, E. O. Aboagye and N. J. Long, *Chem. Sci.*, 2019, **10**, 5603–5615.

5 S. Li, S. Lin, Y. Cheng, T. O. Matsunaga, R. J. Eckersley and M. X. Tang, *Ultrasound Med. Biol.*, 2015, **41**, 1422–1431.
